# Spectrophotometric Measurement of Carbonate Ion in Seawater over a Decade: Dealing with Inconsistencies

**DOI:** 10.1021/acs.est.1c06083

**Published:** 2022-06-07

**Authors:** Elisa F. Guallart, Noelia M. Fajar, Maribel I. García-Ibáñez, Mónica Castaño-Carrera, Rocío Santiago-Doménech, Abed El Rahman Hassoun, Fiz F. Pérez, Regina A. Easley, Marta Álvarez

**Affiliations:** † Centro Oceanográfico de A Coruña (COAC-IEO), CSIC, DC 15001, A Coruña, Spain; ⬢ Institut de Ciències del Mar (ICM), CSIC, DC 08003 Barcelona, Spain; ▼ Instituto de Investigacións Mariñas (IIM), CSIC, DC 36208 Vigo, Spain; ‡ School of Environmental Sciences, University of East Anglia (UEA), Norwich NR47TJ, United Kingdom; § Centro Oceanográfico de Baleares (COB-IEO), CSIC, DC 07015, Palma de Mallorca, Balearic Islands, Spain; ∥ GEOMAR Helmholtz Centre for Ocean Research Kiel, D-24105 Kiel, Germany; ⊥ National Center for Marine Sciences, National Council for Scientific Research in Lebanon (CNRS-L), Beirut, Lebanon; ◆ Chemical Sciences Division, National Institute of Standards and Technology (NIST), DC 20899, Gaithersburg, Maryland, United States

**Keywords:** ocean acidification, saturation states, bio-geochemistry, oceanic carbon cycle, time-series, CO_2_ system monitoring, CO_2_ system variables, climate goal

## Abstract

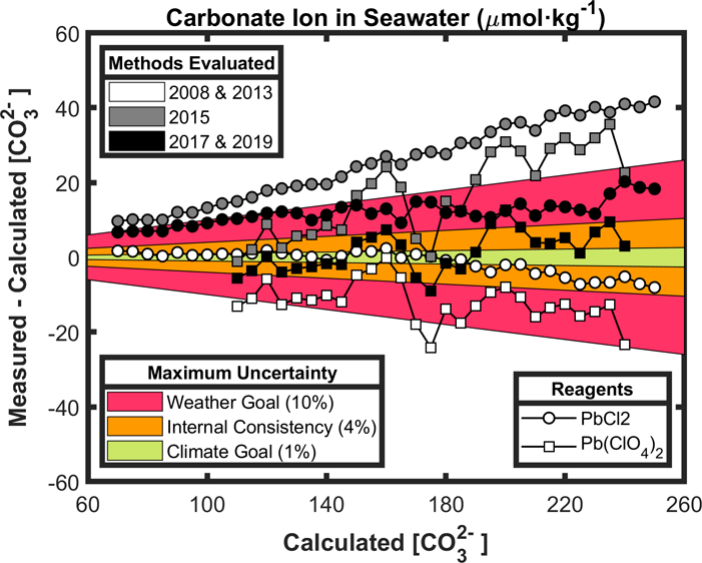

The spectrophotometric methodology for carbonate ion determination in seawater was first published in 2008 and has been continuously evolving in terms of reagents and formulations. Although being fast, relatively simple, affordable, and potentially easy to implement in different platforms and facilities for discrete and autonomous observations, its use is not widespread in the ocean acidification community. This study uses a merged overdetermined CO_2_ system data set (carbonate ion, pH, and alkalinity) obtained from 2009 to 2020 to assess the differences among the five current approaches of the methodology through an internal consistency analysis and discussing the sources of uncertainty. Overall, the results show that none of the approaches meet the climate goal (± 1 % standard uncertainty) for ocean acidification studies for the whole carbonate ion content range in this study but usually fulfill the weather goal (± 10 % standard uncertainty). The inconsistencies observed among approaches compromise the consistency of data sets among regions and through time, highlighting the need for a validated standard operating procedure for spectrophotometric carbonate ion measurements as already available for the other measurable CO_2_ variables.

## 1 Introduction

About a third of the global anthropogenic carbon dioxide (CO_2_) emissions have been absorbed by the global ocean since the preindustrial era.^1^ By acting as a sink of atmospheric CO_2_, the ocean contributes to decreasing the rate at which climate change occurs. Nevertheless, such an effect is counteracted by the resulting increase in seawater acidity (ocean acidification, OA), which determines a decrease in the amount of carbonate ion content in seawater ([CO_3_^2–^]) and therefore of buffering capacity of the ocean.^2−6^

The overall concern for the sustainability of marine life and resources has mobilized the international community to coordinate efforts to track long-term trends in OA by observing seawater CO_2_ variables in coastal^7^ and open ocean^8,9^ time-series. The measurable seawater CO_2_ variables (dissolved inorganic carbon (DIC), total alkalinity (TA), partial pressure of CO_2_ (*p*CO_2_), and pH) are included by the Global Ocean Observing System (GOOS) as Essential Ocean Variables (EOVs) to constrain the CO_2_ system changes and drivers in seawater. Identified and predicted bio-geochemical^10,11^ and ecological^12−14^ OA implications are related to changes in the saturation state (Ω) of seawater for calcium carbonate (CaCO_3_) minerals,^2,11,15^ which control the precipitation and dissolution of its aragonite and calcite forms depending on the available in situ [CO_3_^2–^].^16^ As the oceans acidify, the location and extent of the regions where CaCO_3_ dissolution occurs are expected to increase,^11,15^ particularly in regions with low buffer capacity (Appendix A in Supporting Information).

The increasing amount of CO_2_ data produced demands quality-controlled measurements to ensure their intercomparison. Regarding the four measurable seawater CO_2_ system variables, standard procedures of analysis, data quality control, and reporting are widely established.^17,18^ Additionally, to improve the spatiotemporal resolution of the observation of the seawater CO_2_ system, intensive effort has been made to implement standardized procedures for in situ autonomous measurements performed by autonomous vehicles.^19,20^ Accordingly, relatively simple, fast, and precise automated methods for discrete measurements are encouraged to be implemented.^21^ In this regard, most studies would benefit from moving [CO_3_^2–^] from its category of a derived variable to become the fifth measurable seawater CO_2_ system variable, thus allowing efficient ways of approaching questions relative to CaCO_3_ cycling from [CO_3_^2–^] direct determinations.^22,23^ To this end, Byrne and Yao^24^ first proposed a spectrophotometric method to determine [CO_3_^2–^] in seawater. This method is proposed to be ready for implementation in sustained observations and internal consistency studies.^25,26^ However, its implementation still needs to be validated by independent research groups.

The methodology for the quantification of spectrophotometric [CO_3_^2–^] ([CO_3_^2–^]_spec_) relies on the speciation of lead (Pb(II)) in seawater over a particular pH range (7.7–8.2) at which the complexation of Pb(II) and CO_3_^2–^ predominantly occurs.^27^ The main details on the theory behind the methodology and its evolution are summarized below. A detailed explanation of the motivation for such methodological changes can be found in the Supporting Information (Appendix B) and the related literature.^24,25,27−33^

[CO_3_^2–^]_spec_ is determined by quantifying the ultraviolet light absorbed by lead carbonate, lead chloride/sulfate species, and free Pb^2+^ in Pb(II)-enriched seawater through the following expression:
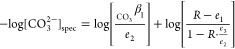
1where _CO_3__β_1_ is the associated equilibrium constant for the complexation of Pb^2+^ and [CO_3_^2–^], and *R* is the absorbance (*A*) ratio of Pb(II) species at 234 nanometers (nm) and 250 nm, corrected for the background absorbance at 350 nm:
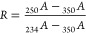
2The molar absorptivity ratios *e*_1_, *e*_2_, and *e*_3_/*e*_2_ are defined as
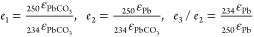
3Equation 1 has the same form as the one used to determine spectrophotometric pH on the total pH scale^34^ and allows quantifying [CO_3_^2–^]_spec_ with a minimum number of parameters via a procedure that closely follows that of spectrophotometric pH. However, unlike other measurable CO_2_ system variables, no recommended standard operating procedure (SOP) has been established for measuring [CO_3_^2–^]_spec_, nor are certified reference materials (CRMs) available for this variable.^17^

The first approach for measuring [CO_3_^2–^]_spec_ was described by Byrne and Yao^24^ in 2008 (BY08). During the following decade, the method was refined by Easley et al.^28^ (EAS13), Patsavas et al.^29^ (PAT15), Sharp et al.^30^ (SHA17), and Sharp and Byrne^25^ (SHA19) in terms of the procedure for obtaining accurate *R* data and, more importantly, the calibration or fitting of the parameters log{_CO_3__β_1_/e_2_}, *e*_1_, and *e*_3_/*e*_2_ (eq 1) needed to relate a particular *R* value (eq 2) with [CO_3_^2–^] (Appendix B).

The former works of BY08 and EAS13 used Pb(II) chloride (PbCl_2_) as the reagent to obtain the *R* measurements. PAT15 proposed a change to Pb(II) perchlorate (Pb(ClO_4_)_2_) and recommended an additional procedure to correct *R* data for sample perturbation due to reagent addition, as
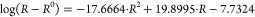
4where *R*^0^ corresponds to the unperturbed *R* value. After SHA17, *R* measurements were no longer corrected with eq 4 but readjusted to include an offset correction for wavelength calibration inaccuracies of the spectrophotometer (*R*^0^), as
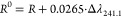
5where Δλ_241.1_ is the spectrophotometer-specific wavelength offset, defined as the difference between the wavelength location of a holmium oxide standard absorbance peak at 241.10 nm specified by the manufacturer minus the wavelength at which the spectrophotometer reports the peak. The sign of eq 5 is reversed with regard to SHA17, where there was an error in the reported equation (J. D. Sharp, personal communication). *R*^0^ refers to the true *R* value for PAT15 and SHA17. Finally, SHA19 followed SHA17 to obtain *R*^0^ but reported the most recent characterization of the terms in eq 1, extending their suitability to a larger range of temperatures and salinities. Before SHA19, the method was solely characterized for use at 25 °C.

All five approaches (Table S1) for [CO_3_^2–^]_spec_ determination are valid for given oceanographic conditions, and none clearly invalidates the others.^24,25,28−30^ In this study, the evolution of the [CO_3_^2–^]_spec_ methodology is evaluated through an internal consistency analysis of a field-based data set obtained during 2009–2020 and expanding over a broad range of oceanographic conditions. A detailed assessment of the different sources of uncertainty in [CO_3_^2–^]_spec_ is discussed. Finally, difficulties found concerning the implementation of the methodology are highlighted.

## 2 Materials and Methods

### 2.1 Cruise Data Compilation

**Figure 1:**
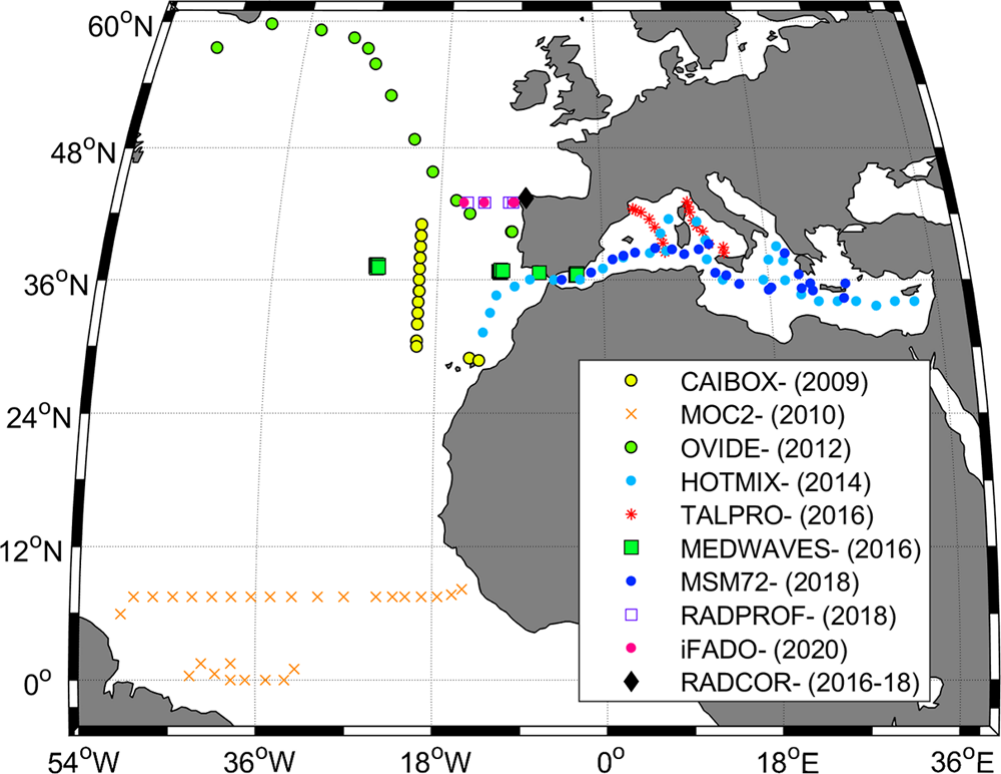
Location of the hydrographic stations of nine open ocean cruises and one coastal time-series site where spectrophotometric carbonate ion content ([CO_3_^2–^]_spec_), pH, TA, and DIC were measured, during 2009–2020. See Table 1 for further details.

Hydrographic and chemical data from nine open ocean cruises in the North Atlantic Ocean and the Mediterranean Sea, and one coastal time-series in the North-East Atlantic Ocean, performed during 2009–2020 (Figure 1 and Table 1), were compiled for assessing the evolution of the [CO_3_^2–^]_spec_ methodology. All data sets include paired measurements of [CO_3_^2–^]_spec_, pH, and TA, and some also include DIC measurements. All seawater CO_2_ system variables were measured following the corresponding SOPs^17^ except [CO_3_^2–^]_spec_ that lacks an SOP. As ancillary data, this study uses hydrographic CTD data and inorganic nutrients (silicate and phosphate). The compiled data set is publicly available in Álvarez et al.^35^

**Table 1:** Cruise Summary Information Containing the Cruise Alias and Assigned EXPOCODE, Research Vessel and Year of Performance, and Ocean Region Studied^a^

Cruise Alias, EXPOCODE	Research vessel, year	Region	Pb(II)reagent(N)	Precision mean ± STD (μmol·kg^–1^) (%)	spectroph. [CO_3_^2–^]_spec_/pH	Salinity range	pH range	TA range (μmol·kg^–1^)	[CO_3_^2–^]_calc_range (μmol·kg^–1^)	TA/DIC range
**CAIBOX,** 29AH20090725	R/V *Sarmiento de Gamboa*, 2009	North East Atlantic	PbCl_2_ (272)	225.0 ± 2.9 (1.3 %)	PE850/SHI2401	34.89–37.08	7.65–8.03	2305–2428	101–227	1.05–1.15
**MOC2**, 29HE20100405	R/V *Hesperides*, 2010	Equatorial Atlantic	PbCl_2_ (625)	249.0 ± 3.4 (1.4 %)	SHI2401	34.50–36.72	7.47–8.13	2294–2408	68–252	1.02–1.18
**HOTMIX,** 29AH20140426	R/V *Meteor*, 2011	Mediterranean Sea	PbCl_2_ (328)	214.0 ± 2.6 (1.2 %)	SHI2600	34.89–39.24	7.72–8.02	2330–2634	118–250	1.07–1.15
**OVIDE,** 29AH20120623	R/V *Sarmiento de Gamboa,* 2012	Subpolar North Atlantic	PbCl_2_ (196)	129.0 ± 1.6 (1.2 %)	PE850	34.46–36.23	7.69–7.99	2292–2398	109–201	1.06–1.13
**TALPRO,** 29AJ20160818	R/V *Angeles Alvariño*, 2016	Western Mediterranean Sea	Pb(ClO_4_)_2_ (115)	211.0 ± 7.0 (4 %)	BK800	37.30–38.82	7.88–8.02	2476–2620	187–242	1.11–1.15
**MEDWAVES**, N/A	R/V *Sarmiento de Gamboa*, 2016	North Eastern Atlantic and Alboran Sea	Pb(ClO_4_)_2_ (148)	131.0 ± 1.0 (0.6 %)	PE850	34.93–38.54	7.70–8.01	2310–2590	111–212	1.06–1.14
**MSM72,** 06M2201S0302	R/V *Maria S. Merian*, 2018	Mediterranean Sea	Pb(ClO_4_)_2_ (294)	238.0 ± 1.0 (0.4 %)	SHI2600	36.45–39.29	7.86–7.99	2397–2639	179–239	1.10–1.14
**RADPROF,** N/A	R/V *Ramon Margalef*, 2018	Iberian Basin North East Atlantic	PbCl_2_ (50)	l31.0 ± 0.8 (0.6 %)	SHI2600	34.90–36.02	7.72–7.97	2316–2383	116–193	1.07–1.13
			Pb(ClO_4_)_2_ (46)	l26.0 ± 2.5 (2 %)						
**RADCOR,** N/A	R/V-*Lura,* 2016 to 2018	North West Galician Coast	PbCl_2_ (128)		SHI2600	33.72–35.74	7.78–8.02	2249–2361	131–200	1.08–1.14
			Pb(ClO_4_)_2_ (74)							
**iFADO,** N/A	R/V *Sarmiento de Gamboa*, 2020	Iberian Basin North East Atlantic	PbCl_2_ (29)	126.0 ± 2.2 (1.7 %)	SHI2600	34.89–36.13	7.72–7.96	2315–2390	115–188	1.07–1.13
			Pb(ClO_4_)_2_ (32)	122.0 ± 1.0 (1 %)						
**iFADO2**, N/A	R/V *Sarmiento de Gamboa*, 2020	Iberian Basin North East Atlantic	PbCl_2_ (33)	127.0 ± 4.9 (3.9 %)	PE850/SHI2600	34.89–36.13	7.72–7.96	2315–2389	115–188	1.07–1.13
			Pb(ClO_4_)_2_ (34)	122.0 ± 2.0 (1.7 %)						

a Information relative to [CO_3_^2–^]_spec_: Pb(II) reagent and number of measurements (*N*); precision in μmol·kg^–1^ and % (mean [CO_3_^2–^]_spec_ ± standard deviation, STD, of the measured replicates is also shown). The spectrophotometer (spectroph.) used for obtaining [CO_3_^2–^]_spec_ and pH is indicated. The ranges of main variables are shown: salinity, pH (total scale at 25 °C), total alkalinity (TA), calculated carbonate ion content ([CO_3_^2–^]_calc_ at 25 °C from pH-TA pair), and TA/DIC ratio (DIC calculated from pH-TA). The reagent PbCl_2_ was 1.1 mmol·L^–1^, and 250 μL (CAIBOX, MOC2, and OVIDE) or 225 μL (rest of cruises) were added. The Pb(ClO_4_)_2_ reagent was 22 mmol·L^–1^, and 20 μL were added.

A solution of unpurified *m*-cresol purple (2 mmol·L^–1^) was used for spectrophotometric pH measurements.^34^ All pH data are reported on the total hydrogen ion scale at 25 °C and atmospheric pressure (hereafter pH). The overall pH precision for all cruises is ± 0.003 pH units based on sample replicates, while the assigned total uncertainty is considered as ± 0.01 pH units (Appendix C).^36−38^ TA samples were measured following a double end-point potentiometric titration,^39−41^ and DIC samples were analyzed through coulometric determination.^42^ TA and DIC accuracies were verified with CRMs.^43^ The TA and DIC precision is ± 2 μmol·kg^–1^ based on sample replicates, and the total uncertainty is ± 3 μmol·kg^–1^ for both TA and DIC.^36^ pH and TA measurements were performed onboard except for the RADCOR time-series, where samples were analyzed at the Instituto Español de Oceanografía (IEO) laboratory the same day and within 2 days after sampling, respectively. DIC measurements were mostly performed postcruise at the IEO laboratory on stored samples poisoned with a saturated solution of mercuric chloride (HgCl_2_), except for the TALPRO and MSM72 cruises during which DIC measurements were performed onboard. Further cruise details are in Table 1.

The overall [CO_3_^2–^]_spec_ measurement procedure has remained the same during the study period. Seawater samples were collected from the Niskin bottles directly into 10 cm quartz cuvettes (∼30 mL volume) that were immediately capped with Teflon caps and heated to 25 °C. All spectrophotometric analyses were performed manually. For each cuvette, a baseline (seawater only) measurement was first performed and followed by the addition of the Pb(II) reagent. Absorbance measurements were recorded in triplicate at three wavelengths (234 nm, 250 nm, and 350 nm) to get an averaged absorbance ratio (*R*; eq 2) for each sample. The temperature of each sample was recorded immediately with a temperature probe (± 0.03 °C) after the absorbance measurements. The Pb(II) reagent, stock concentration, volume addition, and spectrophotometer used for each cruise are detailed in Table 1. Spectrophotometer specifications are detailed in Table S3. Except for CAIBOX and iFADO, pH and [CO_3_^2–^]_spec_ samples were always analyzed with the same spectrophotometer (Table 1). For iFADO, two different spectrophotometers were used onboard for comparison. The precision of the [CO_3_^2–^]_spec_ measurements was evaluated through replicate analysis during each cruise and ranged between ± 1 μmol·kg^–1^ and ± 6.9 μmol·kg^–1^ (± 0.4 % and ± 4 %) (Table 1).

One of the main analytical changes in the methodology reported during the study period implies a change in the Pb(II) reagent from PbCl_2_ (BY08 and EAS13) to Pb(ClO_4_)_2_ (PAT15, SHA17, and SHA19) (Appendix B). In this study, 1666 samples were measured with PbCl_2_ in cruises performed during 2009–2012 (CAIBOX, MOC2, HOTMIX, and OVIDE), and 743 with Pb(ClO_4_)_2_, during 2016–2020 (TALPRO, MEDWAVES, and MSM72). Double measurements with PbCl_2_ and Pb(ClO_4_)_2_ were performed in RADCOR, RADPROF, and iFADO during 2018–2020 (Table 1).

The second major change in the methodology was proposed by SHA17, who recommended readjusting the measured *R* into an offset-corrected *R*^0^ (eq 5 and Appendix B). In this study, the proposed correction was implemented for the cruises where the SHI2600 spectrophotometer was used (Table 1). The equipment was examined for potential wavelength accuracy offsets using a holmium oxide standard (type 667-UV5, provided by Hellma) to assess the Δλ_241.1_ term (eq 5). Lacking a detailed wavelength accuracy test procedure in SHA17, all the measurements were performed reproducing the calibration conditions described in the holmium certification. After 15 determinations, which yielded values between 0.1 nm and 0.3 nm, Δλ_241.1_ was assigned an average value of 0.2 nm, which equals the uncertainty of the certified peak. This offset was applied to *R* data from HOTMIX, MSM72, RADPROF, iFADO, and RADCOR (Table 1). The PE850 spectrophotometer was also examined during the iFADO cruise, with three determinations, yielding Δλ_241.1_ equal to zero. For the remaining cruises, we were unable to examine the equipment, or these had been recalibrated after the cruise; therefore, no wavelength offset correction was applied to CAIBOX, MOC2, OVIDE, TALPRO, and MEDWAVES. Small deviations from the reference temperature (25 °C) were accounted for using SHA19 formulations since the temperature was measured for each sample (mean difference in temperature measurement with regard to 25 °C ranged between 0.02 °C and 0.70 °C among data sets).

*R* values obtained with PbCl_2_ and Pb(ClO_4_)_2_ were indistinctly used with the different formulations in Tables S1 and S2 and [CO_3_^2–^]_spec_ were calculated using all possible combinations between formulations and reagents, under the following assumption: neither the molecular Pb(II) complex added nor its final concentration in the cuvette should affect the absorbance measurements since the method relies on the characterization of the Pb(II) absorbance signal in seawater.^27^ This would be supported by the fact that PAT15 assessed the formulations by BY08 that were obtained with PbCl_2_ with data measured with Pb(ClO_4_)_2_ and proposed using the same formulation for *e*_3_/*e*_2_ as BY08 (Table S2). Hence, five different [CO_3_^2–^]_spec_ values were obtained for each measured *R* value. The BY08, EAS13, PAT15, and SHA17 formulations in Table S2 are referred to 25 °C and atmospheric pressure, being a function of *R* and salinity. Only the formulation by SHA19 is also temperature-dependent. Hereafter, the particular [CO_3_^2–^]_spec_ is noted as [CO_3_^2–^]_specX_, where X is the approach abbreviation (BY08, EAS13, PAT15, SHA17, and SHA19).

Fajar et al.^44^ reported [CO_3_^2–^]_spec_ data from the CAIBOX, MOC2, HOTMIX, and OVIDE cruises, comparing BY08 and EAS13 formulations. This study adds new [CO_3_^2–^]_spec_ data from six cruises in the Mediterranean Sea (TALPRO, MEDWAVES, and MSM72) and the northeast Atlantic Ocean (MEDWAVES, RADPROF, RADCOR, and iFADO). The compiled data cover a wide range of oceanographic conditions in terms of salinity (33.7–39.3), pH (7.47 pH units −8.13 pH units), TA (2249 μmol·kg^–1^ – 2639 μmol·kg^–1^), TA/DIC ratio (1.02–1.18), and the expected [CO_3_^2–^] (68 μmol·kg^–1^ – 252 μmol·kg^–1^) from coastal to open ocean. These ranges are mostly representative of typical open ocean surface conditions over the global ocean (Appendix A; Figure S1).

### 2.2 Definition and Uncertainty in Δ[CO_3_^2–^]

The goodness of the five approaches for quantifying [CO_3_^2–^]_spec_ was evaluated in terms of the internal consistency between measured and calculated [CO_3_^2–^] following the works describing the methodology.^24,25,28−30^ The internal consistency analysis assesses how well predicted values of [CO_3_^2–^] compare to the expected or reference [CO_3_^2–^] values. To this end, [CO_3_^2–^] residuals were obtained as the difference between measured [CO_3_^2–^]_spec_ and [CO_3_^2–^] calculated from thermodynamic equations with paired CO_2_ variables ([CO_3_^2–^]_calc_) (Δ[CO_3_^2–^] = [CO_3_^2–^]_spec_ – [CO_3_^2–^]_calc_; in μmol·kg^–1^). Each of the five [CO_3_^2–^]_spec_ determinations was considered a predicted value, dependent on different sets of calibration functions (Table S2), to be compared to the expected [CO_3_^2–^]_calc_. [CO_3_^2–^]_calc_ was considered the reference value because it is based on paired CO_2_ input variables that have solid SOPs, CRMs exist for some of them, and the methodology for measuring [CO_3_^2–^]_spec_ is itself defined according to [CO_3_^2–^]_calc_ (Appendixes B and D).^24,25,28−30^ As for [CO_3_^2–^]_spec_, corresponding Δ[CO_3_^2–^] will be noted as Δ[CO_3_^2–^]_X_, where X is the approach abbreviation (BY08, EAS13, PAT15, SHA17, and SHA19).

[CO_3_^2–^]_calc_ was estimated using the MATLAB CO2SYS software,^45^ at 25 °C and atmospheric pressure, using both measured pH-TA and TA-DIC pairs, along with total phosphate and silicate. The CO_2_ equilibrium constants of Mehrbach et al.^46^ reformulated on the total hydrogen scale by Lueker et al.^47^ the bisulfate equilibrium constant of Dickson,^48^ and the boron to chlorinity ratio of Lee et al.^49^ were used for the calculations. Absolute differences between [CO_3_^2–^]_calc_ derived from TA-pH and TA-DIC were not significant (Appendix D). Since paired measurements of pH-TA are more abundant than TA-DIC pairs (*N* = 2367 and *N* = 628, respectively), [CO_3_^2–^]_calc_ results shown refer to [CO_3_^2–^]_calc_ from pH and TA.

Studying the internal consistency of Δ[CO_3_^2–^] implies studying how well each of the five predicted values of [CO_3_^2–^]_spec_, dependent on different sets of coefficients (Table S2), compare with the expected [CO_3_^2–^]_calc_ within given limits of uncertainty. A limit of uncertainty of ± 4 % of the expected [CO_3_^2–^]_calc_ was obtained by propagating the standard uncertainties of [CO_3_^2–^]_spec_ and [CO_3_^2–^]_calc_. For [CO_3_^2–^]_spec_, the value of ± 2 % standard uncertainty, assigned among approaches (Table S1 and Appendix C), was considered. The total standard uncertainty of [CO_3_^2–^]_calc_ was calculated using the software package *errors* from Orr et al.^37^ (Appendix D). For the calculations, the following uncorrelated uncertainties were assigned to the input CO_2_ system variables: ± 0.01 pH units for pH, ± 3 μmol·kg^–1^ for TA (section 2.1), and the uncertainty for the equilibrium constants was taken from Table 1 in Orr et al.^37^ Ancillary variables (temperature, salinity, and pressure) and inorganic nutrients were assumed to have negligible standard uncertainty, as in SHA19. The resulting total uncertainty is proportional to [CO_3_^2–^]_calc_ and ranges between 2.5 μmol·kg^–1^ and 8 μmol·kg^–1^ (± 3.2 % – ± 3.7 %) for the studied [CO_3_^2–^]_calc_ range (68 μmol·kg^–1^ to 252 μmol·kg^–1^) (Appendix D; Figure S4). The resulting propagated uncertainty limit to consider Δ[CO_3_^2–^] internally consistent within the [CO_3_^2–^]_calc_ range in this study ranged from ± 3.8 % to ± 4.2 % and was averaged to ± 4 %.

Internally consistent Δ[CO_3_^2–^] values will distribute randomly around zero until the ± 4 % limit. Larger Δ[CO_3_^2–^] values might be due to particularities of each approach, either relative to the respective calibration functions fitting conditions or to the differing methodological recommendations for obtaining *R* values (Table S1). This limit also allows assessing whether there is internal consistency across the five [CO_3_^2–^]_spec_ determinations. Note that the choice of the pH-TA pair implies a more conservative interpretation of the Δ[CO_3_^2–^] with regard to the TA-DIC pair because of the larger uncertainty in [CO_3_^2–^]_calc_ (Appendix D) and is based on assuming quite large uncertainties in the input variables. In addition, Δ[CO_3_^2–^] are also evaluated according to the standard uncertainty limits for weather and climate-quality objectives for OA studies recommended by the Global Ocean Acidification Observing Network (GOA-ON)^50^ of ± 10 % and ± 1 %, respectively.

## 3 Results

### 3.1 Overview of Δ[CO_3_^2–^] Results

**Figure 2:**
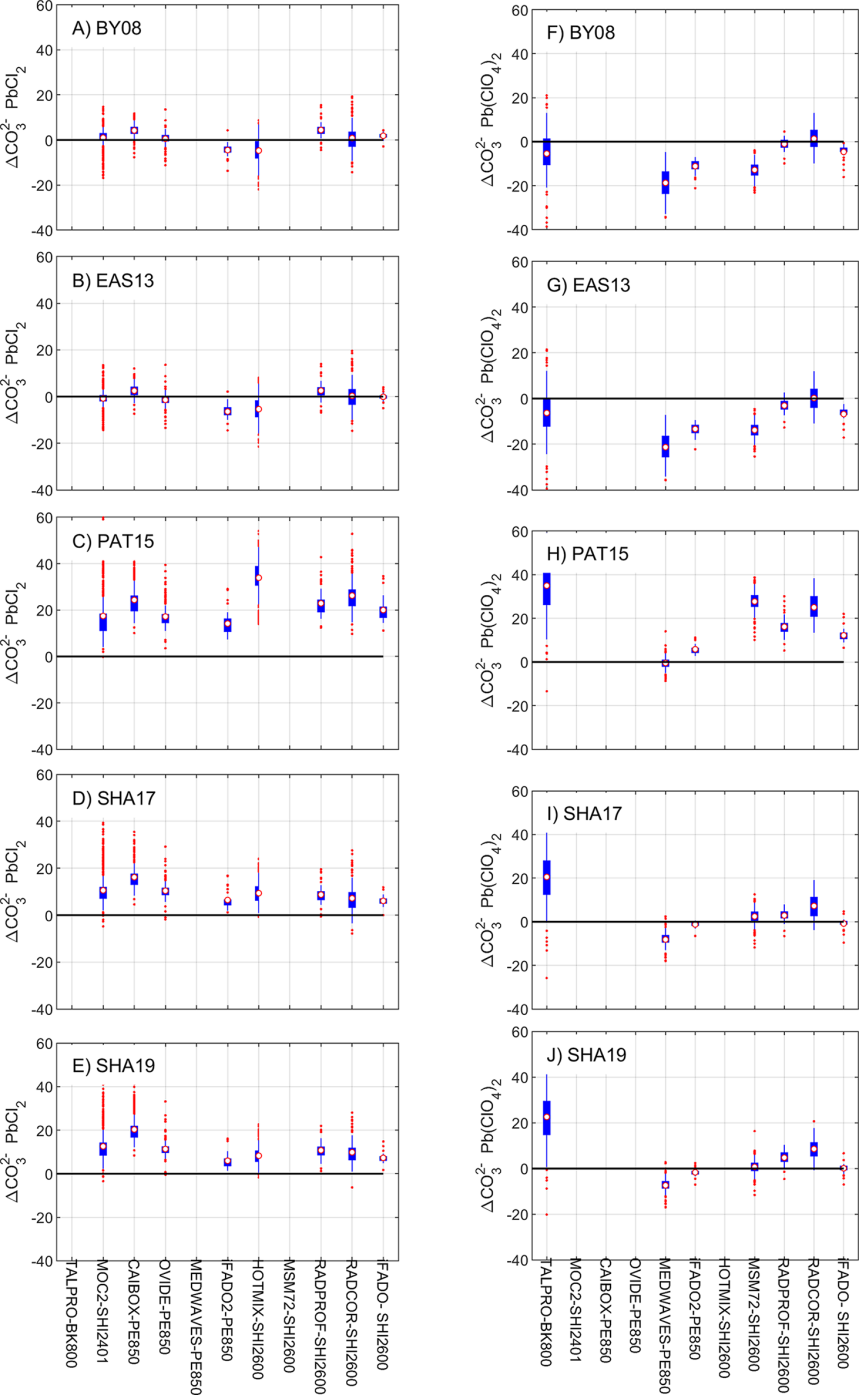
Whisker plots showing spectrophotometric minus calculated carbonate ion content (Δ[CO_3_^2–^] = [CO_3_^2–^]_spec_ – [CO_3_^2–^]_calc_; in μmol·kg^–1^) for cruises in Figure 1 and Table 1. The central dot denotes the mean Δ[CO_3_^2–^], and the lower and upper limits of the blue box are the first and third quartiles, respectively. Whiskers cover 95% of data variance. Red dots are outliers located beyond one-time the interquartile range. [CO_3_^2–^]_spec_ is obtained with five different formulations (Table S2): (A and F) BY08, (B and G) EAS13, (C and H) PAT15, (D and I) SHA17, and (E and J) SHA19. Left and right panels refer to [CO_3_^2–^]_spec_ data measured with PbCl_2_ and Pb(ClO_4_)_2_, respectively. The cruise alias and the spectrophotometer model used (Table 1) are indicated as *x*-axis labels. [CO_3_^2–^]_calc_ is calculated with pH-TA. Calculations are reported at 25 °C and atmospheric pressure except for SHA19 that are reported at the exact temperature of analysis. The SHA17 and SHA19 approaches include a wavelength correction (Δλ_241.1_ = 0.2 nm, Table S1) for cruises where the SHI2600 was used (HOTMIX, MSM72, RADPROF, RADCOR, and iFADO; Table 1) and is null for the remaining cruises.

Figure 2 shows an overview of the Δ[CO_3_^2–^] results by approach, reagent, cruise, and spectrophotometer (values in Table S4). BY08 and EAS13 formulations yield Δ[CO_3_^2–^] close to zero or negative, while the formulations of PAT15, SHA17, and SHA19 yield overall positive Δ[CO_3_^2–^], particularly PAT15. Regardless of the Pb(II) reagent, Δ[CO_3_^2–^] among the five approaches always distribute the same way: Δ[CO_3_^2–^]_EAS13_ and Δ[CO_3_^2–^]_BY08_ are comparable and usually show lower values than Δ[CO_3_^2–^]_SHA17_ and Δ[CO_3_^2–^]_SHA19_ that are also comparable, while Δ[CO_3_^2–^]_PAT15_ shows the highest positive values. In particular, one distinct feature between reagents is that *R* data obtained with PbCl_2_ show larger dispersion and more outliers (red dots) than Pb(ClO_4_)_2_ data.

Δ[CO_3_^2–^]_BY08_ and Δ[CO_3_^2–^]_EAS13_ show mean values close to zero and mostly comprised within the ± 4 % limit for Atlantic Ocean cruises (CAIBOX, MOC2, OVIDE, RADPROF, RADCOR, and iFADO) and one Mediterranean Sea cruise (TALPRO) (Figure 2; Table S4), regardless of the Pb(II) reagent. Negative values beyond the ± 4 % limit are found for one cruise in the Atlantic Ocean (iFADO), two cruises in the Mediterranean Sea (HOTMIX and MSM72), and in regions influenced by Mediterranean waters (MEDWAVES), especially for Pb(ClO_4_)_2_ data (Figure 2; Table S4).

Clearly, Δ[CO_3_^2–^]_PAT15_ shows the largest positive values, mostly above the ± 10 % limit for data from either Pb(II) reagent, except for the MEDWAVES cruise that shows Δ[CO_3_^2–^]_PAT15_ centered around zero (Figure 2; Table S4). Δ[CO_3_^2–^]_SHA17_ and Δ[CO_3_^2–^]_SHA19_ yield mainly positive values between the ± 4 % and ± 10 % limits (Table S4). The SHA17 and SHA19 approaches overall show comparable results, which is expected since all the measurements were performed at 25 °C. These results support the consistency between the SHA17 and SHA19 approaches when the measurements are performed at the same temperature, and differences between them might be due to biases from the reference temperature during analysis (section 2.1), which can be accounted for with the SHA19 approach.

The approaches of SHA17 and SHA19 propose a correction (eq 5) to account for wavelength inaccuracies in the spectrophotometer. In this regard, Δ[CO_3_^2–^]_SHA17_ and Δ[CO_3_^2–^]_SHA19_ from measurements obtained with the SHI2600 spectrophotometer (HOTMIX, MSM72, RADPROF, RADCOR, and iFADO) showed positive values that decreased and approached zero after applying the measured Δλ_241.1_ term for data from either Pb(II) reagent (Figure 2). However, Figure 2 shows that final readjusted Δ[CO_3_^2–^]_SHA17_ and Δ[CO_3_^2–^]_SHA19_ do not center completely around zero for some of these cruises (HOTMIX, RADPROF, and RADCOR). The PE850 spectrophotometer could only be examined during iFADO, for which Δλ_241.1_ was zero, and corresponding Δ[CO_3_^2–^]_SHA17_ and Δ[CO_3_^2–^]_SHA19_ are well centered around zero only for data measured with Pb(ClO_4_)_2_ (Figure 2; Table S4). The Δ[CO_3_^2–^] for the cruises for which the Δλ_241.1_ term could not be measured (CAIBOX, MOC2, OVIDE, TALPRO, and MEDWAVES) approached zero if *R* was readjusted using a Δλ_241.1_ value of about 0.3 nm (results not shown).

### 3.2 Patterns in Δ[CO_3_^2–^] versus [CO_3_^2–^]_calc_

**Figure 3:**
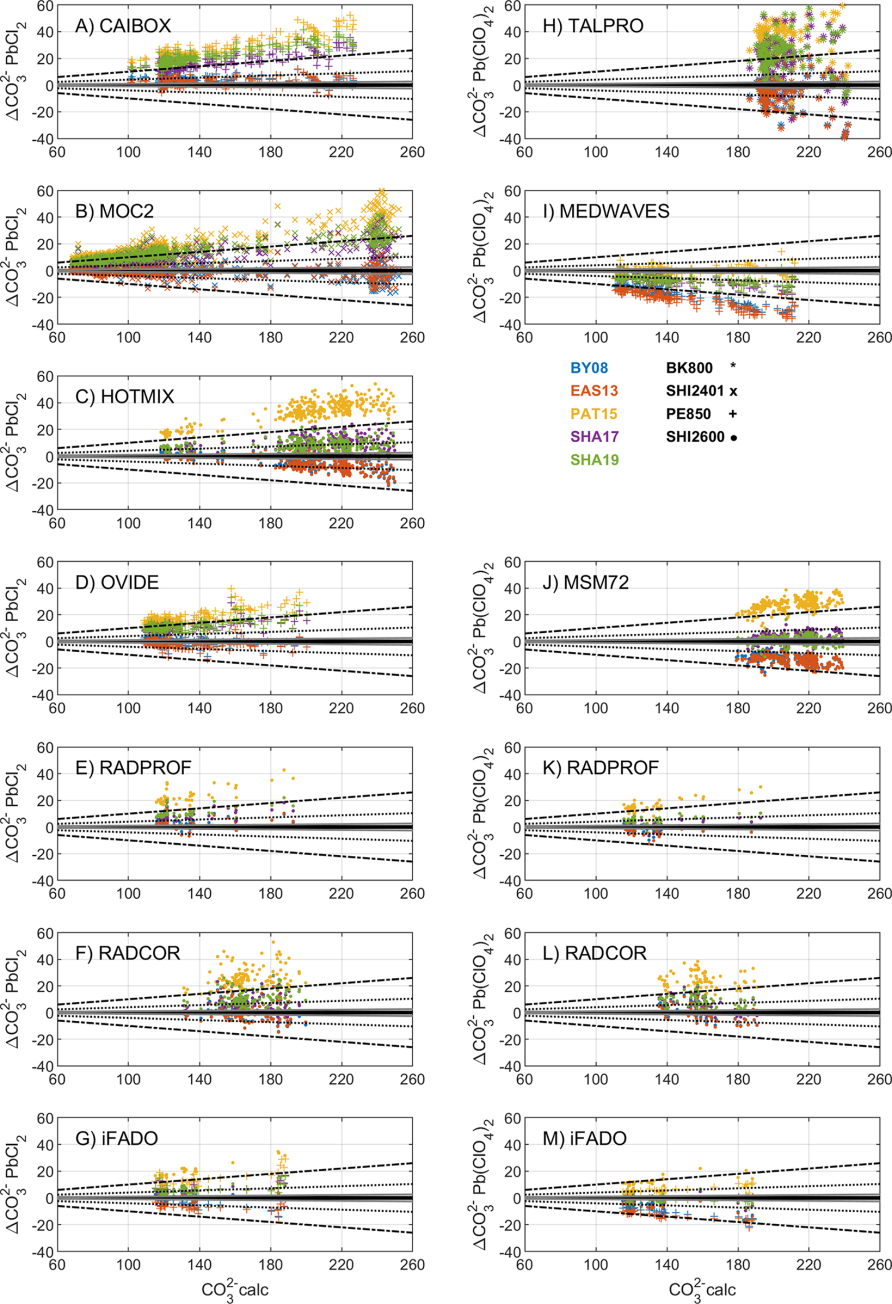
Spectrophotometric minus calculated carbonate ion content (Δ[CO_3_^2–^] = [CO_3_^2–^]_spec_ – [CO_3_^2–^]_calc_; in μmol·kg^–1^) as a function of [CO_3_^2–^]_calc_ (calculated from pH-TA; μmol·kg^–1^) for each cruise (Figure 1; Table 1). [CO_3_^2–^]_spec_ are obtained with five [CO_3_^2–^]_spec_ formulations (Table S1) in color. Spectrophotometer models (Table S3) are identified with different symbols. Panels on the left and the right refer to [CO_3_^2–^]_spec_ data measured with PbCl_2_ and Pb(ClO_4_)_2_, respectively. [CO_3_^2–^]_calc_ are reported at 25 °C and atmospheric pressure, except for SHA19 that are reported at the exact temperature of analysis. The SHA17 and SHA19 approaches include a wavelength correction (Δλ_241.1_ = 0.2 nm; Table S1) for cruises where the SHI2600 was used (HOTMIX, MSM72, RADPROF, RADCOR, and iFADO; Table 1) and is null for the remaining cruises. Gray and dashed lines depict the GOA-ON relative standard uncertainty goals of ± 1 % for the climate-quality objective and ± 10 % for the weather-quality objective, respectively; the dotted line depicts the standard uncertainty limit of ± 4 % attributable to the internal consistency of the data set in this study.

The magnitude of Δ[CO_3_^2–^] is proportional to [CO_3_^2–^]_calc_ itself, as reported in all the works of the methodology,^24,25,28−30,44^ regardless of the Pb(II) reagent, the spectrophotometer, or the approach used (Figure 3). The approaches by BY08 and EAS13 yield Δ[CO_3_^2–^] that mostly scatter around zero (CAIBOX, MOC2, and OVIDE) except for Mediterranean Sea cruises (i.e., high-salinity waters; HOTMIX, MSM72, MEDWAVES, and TALPRO) and for the iFADO cruise, where Δ[CO_3_^2–^]_BY08_ and Δ[CO_3_^2–^]_EAS13_ show negative values that decrease towards higher [CO_3_^2–^]_calc_ (i.e., increasing [CO_3_^2–^]_spec_ underestimation). The approaches by PAT15, SHA17, and SHA19 show the opposite Δ[CO_3_^2–^] patterns, with Δ[CO_3_^2–^]_PAT15_, Δ[CO_3_^2–^]_SHA17_, and Δ[CO_3_^2–^]_SHA19_ that increase proportionally to [CO_3_^2–^]_calc_ (i.e., increasing [CO_3_^2–^]_spec_ overestimation). Figure 3 shows that the same relative distribution among Δ[CO_3_^2–^] from each approach is observed for the whole [CO_3_^2–^]_calc_ range, where Δ[CO_3_^2–^]_EAS13_ ≈ Δ[CO_3_^2–^]_BY08_ < Δ[CO_3_^2–^]_SHA17_ ≈ Δ[CO_3_^2–^]_SHA19_ < Δ[CO_3_^2–^]_PAT15_, as reported in section 3.1. This pattern between approaches was expected for the whole [CO_3_^2–^]_calc_ range at salinity 35, as shown in Figure S2A. It is attributable to differences among calibration functions (Table S2) that are larger at low *R* values (i.e., higher [CO_3_^2–^]_calc_; Figure S2) and higher salinities (Figure S3).

The spectrophotometer used affects the dispersion (Appendix C) in Δ[CO_3_^2–^] (Figure 3). For instance, data from the TALPRO cruise show larger Δ[CO_3_^2–^] dispersion and also larger whisker boxes than other cruises (Figure 2). During TALPRO, [CO_3_^2–^]_spec_ values were measured with a BK800 spectrophotometer (Table 1), which is a single beam and also the least accurate spectrophotometer of all the models used in terms of photometric accuracy (Appendix C; Table S3). In contrast, cruises using the PE850 spectrophotometer (CAIBOX, MEDWAVES, OVIDE, and iFADO) show the lowest Δ[CO_3_^2–^] dispersion for the entire [CO_3_^2–^]_calc_ range (Figure 3). This spectrophotometer has the best photometric accuracy and also a very low value for stray light (Table S3). Between them, in terms of photometric accuracy performance, the SHI2401 and SHI2600 models (Table S3) yield data with increasing dispersion toward higher [CO_3_^2–^]_calc_ for cruises where [CO_3_^2–^]_calc_ > 180 μmol·kg^–1^ (MOC2, HOTMIX, and MSM72). The larger dispersion for RADCOR over the entire [CO_3_^2–^]_calc_ range might be related to being a coastal site with higher inherent variability.^7^

Also, the spectrophotometer used affects the overall bias (Appendix C) in Δ[CO_3_^2–^]. PE850 exemplifies well that highly precise equipment in terms of dispersion (i.e., photometric accuracy; Table S3) can exhibit very different performance in terms of bias (i.e., wavelength accuracy; CAIBOX, MEDWAVES, OVIDE, and iFADO; Figure 3). In this regard, since Δ[CO_3_^2–^] from each of the five approaches always keep the same relationship among them, the approaches that respectively show the most internally consistent Δ[CO_3_^2–^], within the ± 4 % limit of [CO_3_^2–^]_calc_, are cruise-dependent, in relation to the spectrophotometer bias: SHA17 and SHA19 (MSM72, Pb(ClO_4_)_2_; iFADO, both reagents), EAS13 and BY08 (CAIBOX, MOC2, and OVIDE, all using PbCl_2_; RADPROF and RADCOR, both reagents), and PAT15 (MEDWAVES, Pb(ClO_4_)_2_).

### 3.3 Limits of Consistency for Δ[CO_3_^2–^]

No approach fulfills the GOA-ON relative standard uncertainty goal of ± 1 % for the climate-quality objective, but most results, except those obtained with the PAT15 approach, meet the weather-quality objective of ± 10 % uncertainty for any range of [CO_3_^2–^]_calc_ (Figure 3; Table S5).

Cruises in the Atlantic Ocean, except the iFADO cruise, show Δ[CO_3_^2–^] values within ± 4 % uncertainty with the BY08 and EAS13 approaches for the whole [CO_3_^2–^]_calc_ range (Figure 3; Table S5). Hence, [CO_3_^2–^]_specBY08_ or [CO_3_^2–^]_specEAS13_ are internally consistent within the assumed uncertainty (Section 2.2). This was already reported by Fajar et al.^44^ for the Atlantic Ocean data (CAIBOX, MOC2, and OVIDE). Although the EAS13 approach was reported to be accurate enough (± 2 %) only below 180 μmol·kg^–1^ by PAT15,^29^ the results in this study suggest that both BY08 and EAS13 formulations determine consistent [CO_3_^2–^]_spec_ (± 4 %) in the North Atlantic Ocean, over the 68 μmol·kg^–1^ – 252 μmol·kg^–1^ range.

For high-salinity waters, the BY08 and EAS13 approaches yield mostly negative Δ[CO_3_^2–^] (although still within ± 4 % when using PbCl_2_ (HOTMIX)) that show larger Δ[CO_3_^2–^] (within ±10 % or beyond) when using Pb(ClO_4_)_2_ (TALPRO, MEDWAVES, and MSM72; Table S5). The EAS13 approach was reported to underestimate [CO_3_^2–^]_spec_ at salinity > 36, [CO_3_^2–^]_calc_ > 150 μmol·kg^–1^, and high pH.^28,44^ This study shows that the underestimation of [CO_3_^2–^]_spec_ occurs for the HOTMIX, MEDWAVES, and MSM72 cruises, with the BY08 and EAS13 approaches. In this regard, EAS13 suggested that the Pb(II) complexation model should be extended to include the formation of other potential Pb(II) complexes to address the trend in the residuals seen at high [CO_3_^2–^] (Appendix B). However, opposite to the approaches of BY08 and EAS13, the most recent approaches of SHA17 and SHA19, with the latter covering a wide salinity fitting range (Table S1), do not underestimate [CO_3_^2–^]_spec_; yet, in some cruises overestimate it (Figure 3; Table S5). Hence, the hypothesis of a mischaracterization of the Pb(II) complexation in high-salinity and high-pH waters would not be supported. Instead, the fitting procedure for the terms in eq 1 for each approach might cause the observed negative Δ[CO_3_^2–^], as the greatest changes regarding the stability constant for the formation of the PbCO_3_ complex (Figure S2B) and the terms  and  (Figure S3) were mostly generated for high-salinity and low *R* values.

## 4 Discussion

Both the observed mean Δ[CO_3_^2–^] values and corresponding trends versus [CO_3_^2–^]_calc_, taking into account reagents, equipment specifications and calibration, salinity ranges, and [CO_3_^2–^]_calc_, suggest that the method is inconsistent among approaches with regard to its performance for [CO_3_^2–^]_spec_ measurement. In this section, the methodological changes among approaches are assessed to identify the factors that explain the observed differences in Δ[CO_3_^2–^].

### 4.1 Random and Systematic Uncertainty of the Absorbance Ratio (*R*): Precision and Accuracy of Absorbance Measurements

Since *R* values are inversely proportional to [CO_3_^2–^]_spec_ (i.e., lower *R* values yield higher [CO_3_^2–^]_spec_; Figure S2A inset), the assessment of [CO_3_^2–^]_spec_ is less precise at a higher [CO_3_^2–^], and this is independent of the equipment or approach used. Within *R* (eq 2), _250_*A* varies substantially with pH,^24,27,33^ becoming lower as [CO_3_^2–^] increases, while _234_*A* is always higher, lying close to the isosbestic point (Appendix C) and is thus less sensitive to changes in [CO_3_^2–^]. In consequence, lower *R* values will inherently have larger random uncertainty, or lower precision, which translates into a fan-shape distribution of Δ[CO_3_^2–^] for results obtained with any approach, particularly at [CO_3_^2–^]_calc_ > 180 μmol·kg^–1^ (Figure 3). This fact, derived from *R* being a ratio of absorbances with dissimilar magnitudes, can be empirically evidenced using a Monte Carlo analysis (Appendix E; Figure S5).

Technical specifications of the spectrophotometer regarding the photometric and wavelength accuracies, and the stray light (Table S3) might be critical for setting the mean Δ[CO_3_^2–^] value and its relationship to [CO_3_^2–^]_calc_. In this study, the spectrophotometers used have variable values for photometric accuracy (Appendix C), which translates into variable *R* uncertainty (Table S3) that will affect Δ[CO_3_^2–^] to a different extent but always cause the same distribution (Figure S5). A spectrophotometer with low photometric accuracy will introduce more random noise in *R* measurements, especially at low *R* values, resulting in even more dispersed Δ[CO_3_^2–^] at high [CO_3_^2–^]. Another factor affecting random uncertainty might be the stray light of the spectrophotometer (Appendix C). By combining both random sources of uncertainty and according to the spectrophotometers specifications (Table S3), the spectrophotometer models could be ordered from the highest to the lowest expected precision as PE850, SHI2600, SHI2401, and BK800, which agrees with the observed results (Figure 3; Tables S4 and S5).

While random uncertainty in *R* explains the larger dispersion of Δ[CO_3_^2–^] at high [CO_3_^2–^], it does not explain systematic biases in Δ[CO_3_^2–^] toward positive or negative values outside the ± 4 % limit (Figure 3). The wavelength accuracy is critical in this regard (Appendix C) since it could be significant when measuring _250_*A*, located on a slope, where small variations in wavelength cause significant changes in _250_*A*, and to a lesser extent in _234_*A*, located near a peak. This was accounted for in SHA17, who proposed a wavelength offset term (Δλ_241.1_; eq 5) to correct inaccuracies in the equipment wavelength calibration.

**Figure 4:**
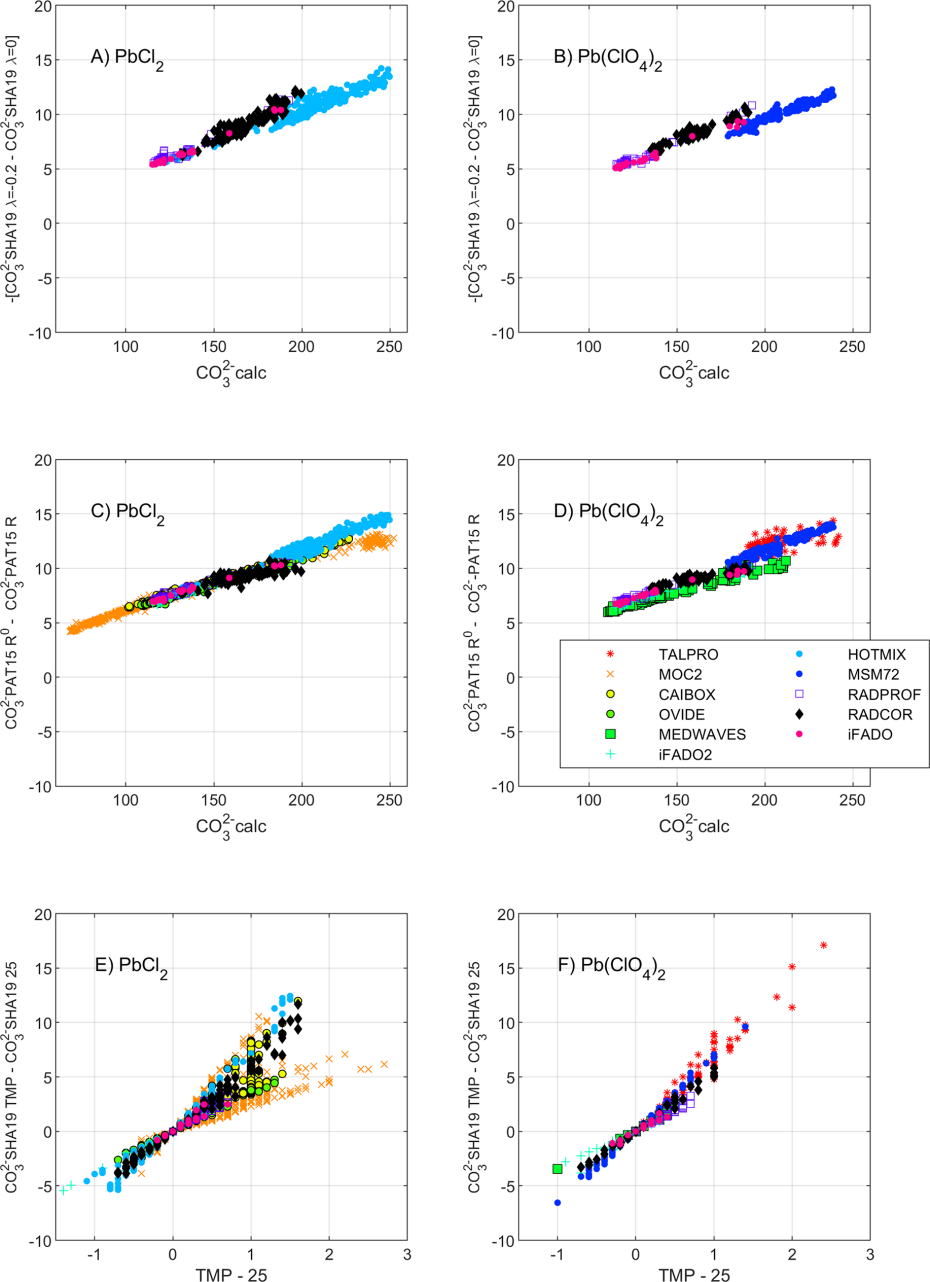
Effect on [CO_3_^2–^]_spec_ (in μmol·kg^–1^) of (A and B) the wavelength offset correction proposed by SHA17 as a function of [CO_3_^2–^]_calc_ (calculated from TA-pH; μmol·kg^–1^), (C and D) the Pb(II) reagent perturbation correction according to PAT15 as a function of [CO_3_^2–^]_calc_, and (E and F) the functions proposed by SHA19 as a function of the temperature bias regarding 25 °C. Differences are calculated as (A and B) [CO_3_^2–^]_spec_ calculated using a wavelength offset correction (Δλ_241.1_) of 0.2 nm (eq 4) minus [CO_3_^2–^]_spec_ obtained without correction, for cruises using the SHI2600 spectrophotometer; (C and D) [CO_3_^2–^]_spec_ calculated with the corrected minus the original Pb(II) absorbance ratio (*R*^0^ and *R*, respectively; Table S1); (E and F) [CO_3_^2–^]_spec_ at the real temperature (TMP) of analysis minus [CO_3_^2–^]_spec_ at 25 °C. Data measured with PbCl_2_ or Pb(ClO_4_)_2_ are shown on the left and the right panels, respectively. All panels share the same legend.

In this regard, measuring the holmium oxide standard to assess Δλ_241.1_ for the SHI2600 spectrophotometer was not simple. The Δλ_241.1_ term is supposed to remain constant as long as the equipment is not recalibrated.^30^ However, 15 Δλ_241.1_ measurements were averaged to obtain a stable value (section 2.1). Our experience quantifying Δλ_241.1_ indicates that (i) according to SHA17, shifts in Δλ_241.1_ on the order of 0.05 nm are supposed to be significant, which would imply that the holmium standard should be measured each time that the spectrophotometer is used, not only when recalibrated, because differences of this order were found for measurements of the same holmium oxide standard in different measurement exercises; (ii) the average Δλ_241.1_ found for SHI2600 (0.20 nm ± 0.06 nm) is lower than the wavelength accuracy of the spectrophotometer (± 0.3 nm; Table S3), and (iii) the average Δλ_241.1_ equals the uncertainty of the certified holmium peak position (241.15 nm ± 0.20 nm). Therefore, *R* measurements are likely corrected over the limits of the SHI2600 spectrophotometer and holmium standard specifications. However, when the offset correction is applied, Δ[CO_3_^2–^]_SHA19_ decrease proportionally to [CO_3_^2–^]_calc_ by about 5 μmol·kg^–1^ to 14 μmol·kg^–1^, for measurements with both reagents (Figure 4A, B). The SHA19 approach also uses Δλ_241.1_. Despite adding complexity to the analysis procedure, both Δ[CO_3_^2–^]_SHA17_ and Δ[CO_3_^2–^]_SHA19_ meet the ± 4 % uncertainty limit (Tables S3 and S4) and can be considered internally consistent, when the Δλ_241.1_ term is assessed, with the HOTMIX and RADCOR exceptions.

Technical specifications of the spectrophotometer seem crucial for precise and accurate *R* measurements. Most equipment might not achieve the accuracy necessary for [CO_3_^2–^]_spec_ determination (Table S3), including the model UV8453 used for describing the methodology (Table 1).^30^ An accurate quantification of *e*_3_/*e*_2_ and the fitting of  in eq 1 would benefit from using the best equipment in terms of technical specifications.

### 4.2 Change in Pb(II) Reagent and Double Addition Correction

PAT15 proposed a change in the Pb(II) reagent, from PbCl_2_ to Pb(ClO_4_)_2_, doubling the final Pb(II) concentration in the cuvette (Table S1), to increase the signal-to-noise ratio of the absorbance measurements. Additionally, the authors proposed an *R* perturbation correction (eq 4) that enlarges the resulting [CO_3_^2–^]_specPAT15_ proportionally to [CO_3_^2–^]_calc_ by 5 μmol·kg^–1^ to 15 μmol·kg^–1^ with regard to uncorrected *R* values (Figure 4C, D). As a result, Δ[CO_3_^2–^]_PAT15_ values reach 20 μmol·kg^–1^ – 40 μmol·kg^–1^ at higher [CO_3_^2–^]_calc_ (Figure 3). If the correction is not applied, Δ[CO_3_^2–^]_PAT15_ compares well with Δ[CO_3_^2–^]_SHA17_ and Δ[CO_3_^2–^]_SHA19_ (data not shown), which confirms that the large positive Δ[CO_3_^2–^]_PAT15_ values are mainly caused by the perturbation correction. In this regard, no *R* perturbation correction was reported by BY08 or EAS13 for PbCl_2_ since the Pb(II) concentration in the cuvette (7.5 μmol·L^–1^; Table S1) does not induce significant sample perturbation.^28^ Neither of the most recent procedures by SHA17 and SHA19 recommends using a perturbation correction for *R* values measured with Pb(ClO_4_)_2_.

**Figure 5:**
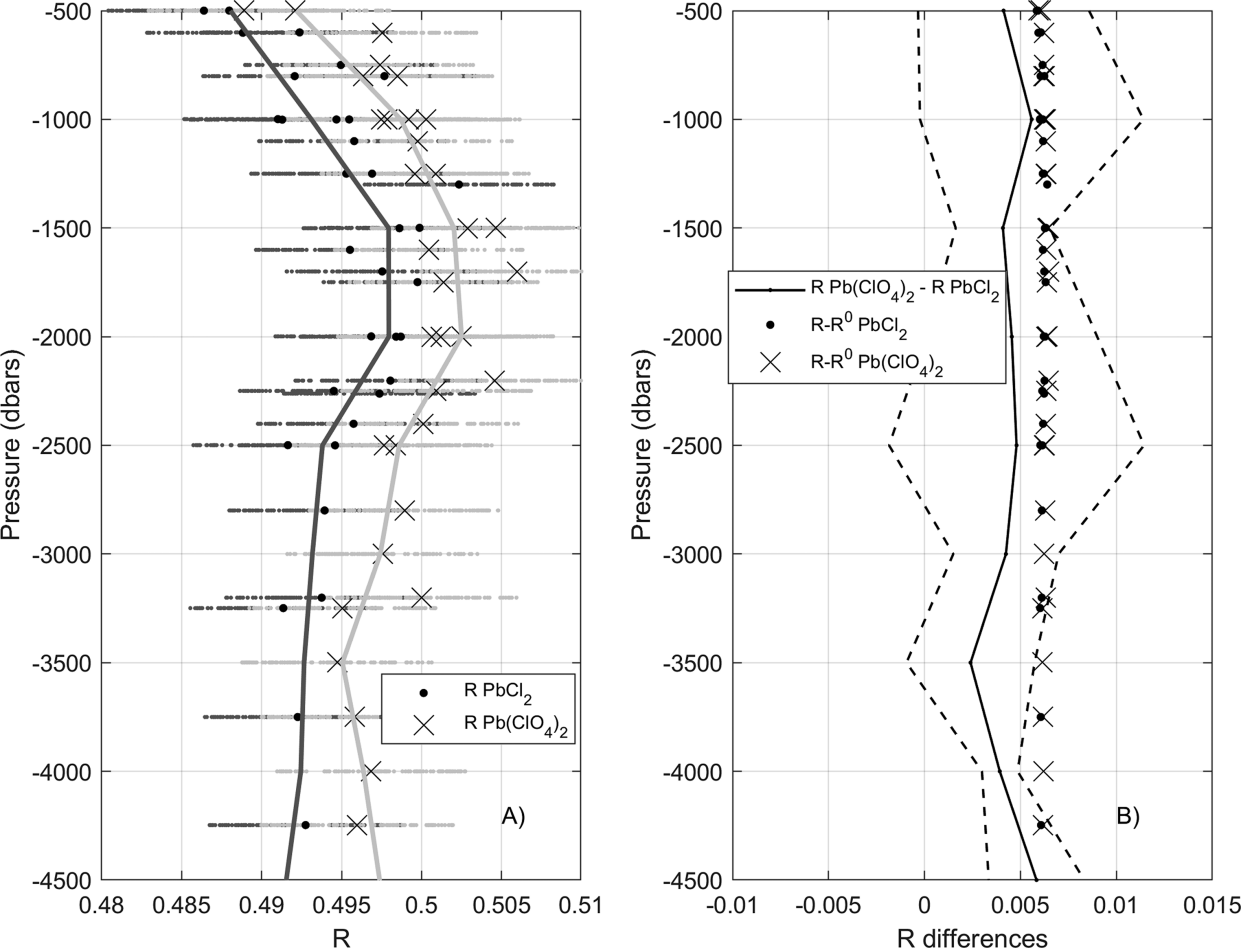
(A) Mean profiles of Pb(II) absorbance ratios (*R*) for sample replicates measured with PbCl_2_ (dark line and dots) and Pb(ClO_4_)_2_ (gray line and crosses) during the RADPROF cruise (Table 1). Horizontal dotted lines accompanying each *R* value correspond to a Monte Carlo perturbation analysis introducing a random uncertainty within ± 0.006 in measured *R* values according to SHI2600 specifications (Table S3). (B) Mean difference profile of *R* measured with Pb(ClO_4_)_2_ minus *R* measured with PbCl_2_ (solid line) and corresponding uncertainty (dashed lines) calculated considering the Monte Carlo perturbed *R* results shown in panel A. Additionally, the value of the *R* perturbation correction according to PAT15 (*R*^0^ – *R*; Table S1), for RADPROF replicate measurements with PbCl_2_ (dark dots) and Pb(ClO_4_)_2_ (gray crosses) is also depicted. The sign of the perturbation values has been reversed for representation purposes.

During the RADPROF cruise, *R* values were measured on replicate samples with PbCl_2_ and Pb(ClO_4_)_2_ with the SHI2600 spectrophotometer (Table 1). The same comparison experiment was repeated during the iFADO cruise, where two different spectrophotometers were used: SHI2600 and PE850 (Table 1). RADPROF results reveal that *R* values measured with Pb(ClO_4_)_2_ are higher than those measured with PbCl_2_ (Figure 5A) for sample replicates. The difference is almost constant for the whole depth profile and amounts to 0.0044 ± 0.0010 (Pb(ClO_4_)_2_ – PbCl_2_; Figure 5B). Noticeably, the two reagents might behave differently characterizing the Pb(II) absorbance signal for the same seawater conditions. Considering a random uncertainty of ± 0.006 (Table S3) in *R* measurements, according to SHI2600 specifications, the mean *R* difference found between reagents is close to the *R* random uncertainty. In this regard, a Monte Carlo experiment introducing this random noise in the bulk *R* data from both reagents shows that resulting *R* values overlap (Figure 5A). This overlapping is magnified for spectrophotometers with lower photometric accuracy (data not shown). This would explain the absence of a significant difference of mean Δ[CO_3_^2–^] using either reagent and formulations (Figure 2 and Table S4). In addition, Figure 5B shows that the observed *R* difference between reagents has almost the same magnitude but opposite sign to the magnitude of the *R* perturbation correction proposed by PAT15 (−0.006 ± 0.0005) for data measured using PbCl_2_ or Pb(ClO_4_)_2_. During the iFADO cruise, the same overall results were observed for both the SHI2600 and PE850 spectrophotometers: *R* differences between reagents (Pb(ClO_4_)_2_ – PbCl_2_) were 0.0050 ± 0.0015 and 0.0061 ± 0.0006, respectively (data not shown). In this regard, the possibility that perchlorate (ClO_4_^–^) could be absorbing light at the target wavelengths was examined using a solution of ClO_4_^–^ (>98% purity) in different seawater conditions in terms of salinity and [CO_3_^2–^]_calc_, showing that measured absorbances were not different from zero at the target wavelengths (results not shown).

During the iFADO cruise, further comparison experiments of reagents and spectrophotometers were performed on quadruplicate samples through scan measurements (Appendix F). The observed results show that _234_*A* and _250_*A* are always higher when measured using Pb(ClO_4_)_2_ compared to PbCl_2_ (Figure S6), which is expected considering that the final Pb(II) concentration in the cuvette is higher when using Pb(ClO_4_)_2_ (Table S1). _234_*A* and _250_*A* measurements obtained with different spectrophotometers are broadly coincident for data measured with PbCl_2_ (Figure S6A,B), which is also the case for _250_*A* data measured with Pb(ClO_4_)_2_ (Figure S6D). While a good agreement was observed between the SHI2600 and PE850 spectrophotometers for the measurements of _234_*A* and _250_*A* using PbCl_2_ (Figure S6A,B), only measurements of _250_*A* agreed between the two instruments when Pb(ClO_4_)_2_ was used (Figure S6D). In fact, _234_*A* values measured with Pb(ClO_4_)_2_ clearly deviate from the 1:1 line, with SHI2600 yielding higher values than PE850 (Figure S6C). These results are well depicted in Figure S7A, showing the overlapping of _250_*A* versus _234_*A* measured with PbCl_2_ for both spectrophotometers. However, Figure S7B also shows that Pb(ClO_4_)_2_ data split into two groups depending on the spectrophotometer used. Consequently, *R* values from PbCl_2_ are comparable between SHI2600 and PE850, but PE850 yields higher *R* values when using Pb(ClO_4_)_2_ (Figure S7C).

The observed results are surprising as larger differences in the _250_*A* value were expected because it is located in a slope area. No sampling, preservation, or manipulation differences between replicates can be ascribed to the observed results. Considering the similar technical specifications between SHI2600 and PE850 in terms of wavelength and photometric accuracy (Table S3), and the fact that mean _234_*A* and _250_*A* integrate absorbance data around ± 2 nm of the target wavelengths (Appendix F), the only difference between them is the stray light specification (Appendix C). However, this would lead to _234_*A* PE850 > _234_*A* SHI2600, which is opposite to the observed results.

In conclusion, the change in Pb(II) reagent seems to affect the determination of [CO_3_^2–^]_spec_. Using Pb(ClO_4_)_2_ improves data dispersion (Figure 2) regarding PbCl_2_, but Pb(ClO_4_)_2_ also appears to be more sensitive to inaccuracies in the absorbance signal related to the technical specifications of the equipment.

### 4.3 In Situ Temperature Correction

The approaches by BY08, EAS13, PAT15, and SHA17 need temperature control at 25 °C ± 0.05 °C (Table S1), while SHA19 introduced temperature-dependent terms in the absorptivity coefficients and in the formation constant (Table S2) that allow determinations over a range of temperatures (3 °C < *t* ± 0.05 °C < 40 °C; Table S1). Recall that Soli et al.^31^ reported that the formation constant (eq S3) is almost constant for the range 15–35 °C. This is reflected in Table S2 where the temperature dependence of the formation constant is minor, while the absorptivity terms have stronger temperature dependence. SHA19 reported an error of about 3% in measured [CO_3_^2–^]_specSHA19_ for 1 °C bias in temperature. Using the SHA19 approach and the temperature recordings for each sample, the observed bias in [CO_3_^2–^]_spec_ for samples that lacked temperature control at 25 °C (Figure 4E, F) is in agreement with the bias reported by SHA19. Hence, [CO_3_^2–^]_spec_ is underestimated in samples with a temperature higher than 25 °C that are incorrectly reported at 25 °C, underestimating [CO_3_^2–^]_spec_ by about 5 μmol·kg^–1^ (≈ 3%) for 1 °C positive bias in temperature. All the approaches recommend temperature control within ± 0.05 °C (Table S1), and the results of this study suggest that temperature control is needed at least within ± 1 °C to reduce uncertainty within the ± 4 % limit.

## 5 Conclusions

The assessment of the internal consistency between [CO_3_^2–^]_spec_ from the five different approaches of the methodology and [CO_3_^2–^]_calc_ according to data in this study allowed a detailed comparison of the differential factors among the evolving approaches studied, regarding the respective sets of calibration functions to infer [CO_3_^2–^]_spec_ (Table S2) as well as an examination of other methodological updates proposed through time (Table S1). Overall results suggest that further documentation is needed until [CO_3_^2–^]_spec_ can be implemented as the fifth measurable variable of the seawater CO_2_ system. A summary of areas where improvement is needed to enhance the long-term reproducibility of [CO_3_^2–^]_spec_ to warrant the broader implementation of [CO_3_^2–^]_spec_ measurements are detailed in the following:

(i) Current robustness of [CO_3_^2**–**^]_spec_ for OA monitoring studies. [CO_3_^2–^]_spec_ observations reported in this study suggest that approaches of the methodology assessed (BY08, EAS13, PAT15, SHA17, and SHA19; Table S1) are not yet ready for applications that require climate-quality measurements because they do not meet the GOA-ON objective of ± 1 % standard uncertainty for the whole studied range of [CO_3_^2–^]_calc_. Moreover, none of the five approaches assessed is clearly the best option for being globally implemented. But all the approaches except PAT15 fulfill the weather-quality standard of ± 10 %. Those approaches proven to meet the weather-quality objective could be used and further tested in studies facing large temporal or spatial variability in [CO_3_^2–^] or in other compatible applications like OA experimentation in aquaria, shellfish aquaculture, or coastal conservation.
(ii) [CO_3_^2**–**^]_spec_ as the fifth CO_**2**_ measurable variable and internal consistency analysis of CO_**2**_ overdetermined systems. Few [CO_3_^2–^]_spec_ observations reported in this study meet the expected ± 2 % standard uncertainty attained in reference bibliography (Table S1). Furthermore, the relative Δ[CO_3_^2–^] distribution between approaches remains the same for the entire salinity and [CO_3_^2–^]_calc_ ranges studied, where [CO_3_^2–^]_specBY08_ ≈ [CO_3_^2–^]_specEAS13_ < [CO_3_^2–^]_specSHA17_ ≈ [CO_3_^2–^]_specSHA19_ < [CO_3_^2–^]_specPAT15_ but demonstrates an overall lack of consistency between them. This implies that using diverse *R* data sets with newer algorithms does not guarantee an improvement in the recalculated data set, which is particularly unfavorable for the maintenance of time-series. Time-series or data sets tracking the [CO_3_^2–^]_spec_ methodological evolution (Table S1) would lack consistency between results obtained with former procedures (BY08 and EAS13) with regard to the most recent approaches from SHA17, SHA19, and PAT15. According to these findings, it might not be recommendable to include [CO_3_^2–^]_spec_ as the fifth CO_2_ measurable variable until the methodology is extensively reviewed and implemented by more research groups. There are few references from independent groups reporting the use of the [CO_3_^2–^]_spec_ methodology,^44^ which is a clear disadvantage for its widespread implementation as a well-tested method. This point was considered in the GLODAPv2.2020 update where no [CO_3_^2–^] data were included.^36^ The inclusion of [CO_3_^2–^]_spec_ as the fifth measurable variable with any of the approaches for internal consistency studies would likely add noise in the already complicated seawater CO_2_ system modeling and characterization, where known inconsistencies are currently under debate.^38,51^ Additionally, caution is needed when pairing [CO_3_^2–^]_spec_ with another seawater CO_2_ variable to estimate the whole seawater CO_2_ system.^25^ According to the observed results, a standard uncertainty larger than 2 % should be assigned to [CO_3_^2–^]_spec_ to evaluate propagated uncertainties in derived CO_2_ variables.
(iii) SOP for [CO_3_^2–^]_spec_ measurements and factors to be further specified. This study highlights several inconsistencies in the [CO_3_^2–^]_spec_ methodology evolution related to the Pb(II) reagent, the equipment specifications, and controversial perturbation and wavelength offset corrections, which points to the need to provide a well-described SOP for end-users, as available for the other four seawater CO_2_ system variables^17^ that allows the successful performance of the method in diverse seawater conditions. A summary of likely significant factors that should be revisited and made clearer to users follows.

### 5.1 Spectrophotometer

The technical specifications regarding photometric and wavelength accuracies and the stray light seem crucial for [CO_3_^2–^]_spec_ determination. The observed Δ[CO_3_^2–^] values reflect the dependence between the degree of data dispersion and the photometric accuracy of the spectrophotometer. The stray light seems to cause significant differences in _234_*A* between spectrophotometers when Pb(ClO_4_)_2_ is used, but the reason is unknown. Additionally, the use of this reagent demands the assessment of an offset correction (Δλ_241.1_; Table S1) related to the wavelength accuracy of the equipment to correct systematic biases in the data. However, Δλ_241.1_ assessment and implementation should be described more clearly since it seems controversial, going beyond the limits of uncertainty of both the wavelength accuracy specifications of the spectrophotometers and the holmium oxide certified standard accuracy.

### 5.2 Pb(II) Reagent

*R* values obtained with Pb(ClO_4_)_2_ are about 0.004–0.006 units higher than *R* measured with PbCl_2_. Such difference seems insignificant when introduced in the formulations of the different approaches for calculating [CO_3_^2–^]_spec_. In this regard, Δ[CO_3_^2–^] showed less dispersion for data measured with Pb(ClO_4_)_2_, which would support the reagent change. Nevertheless, Pb(ClO_4_)_2_ might cause the _234_*A* characterization to become more sensitive to the spectrophotometer specifications, thus impacting *R* measurements to a larger extent than when using PbCl_2_. Differences in *R* values measured with the two Pb(II) reagents should be assessed to ensure the production of consistent data sets between groups and in time.

### 5.3 Temperature Sensitivity

Temperature control is needed within ± 0.05 °C to obtain [CO_3_^2–^]_spec_ as accurate as possible within the capabilities of the methodology or at least within ± 1 °C to keep the uncertainty within the ± 4 % limit attributable to internal consistency.

### 5.4 Ranges of Salinity and pH Validity

The salinity ranges assessed to fit the various sets of calibration functions might impact the accuracy of the resulting [CO_3_^2–^]_spec_ and the applicability of a given approach. Valid salinity ranges for the BY08 and EAS13 approaches comprise the Atlantic Ocean but not high-salinity waters (*S* > 36.5) where they underestimate [CO_3_^2–^]_spec_. Overestimation of [CO_3_^2–^]_spec_ is observed, for both the Atlantic Ocean and the Mediterranean Sea with the approaches of PAT15, SHA17, and SHA19. The approaches of BY08 and SHA19 characterized the calibration functions for large salinity ranges: 20 < *S* < 36 and 20 < *S* < 40, respectively (Table S1). However, these salinity ranges were reached through modification of natural seawater in the laboratory. In BY08 all data was experimental, while in SHA19 they used data from natural samples but merged them with experimental data with broader salinity conditions. The salinity range assessed with natural seawater samples for characterizing the sets of calibration functions is shorter: 26.6 < *S* < 36.72, among approaches (Table S1). Although the five sets of calibration functions showed the lowest [CO_3_^2–^]_spec_ uncertainties at the lower ranges of [CO_3_^2–^]_calc_ (Figures 3 and S5), suggesting that this would likely apply for seawater at lower salinities than the lowest ranges in this study (i.e., *S* < 33.7), observational evidence is needed to confirm this. Further [CO_3_^2–^]_spec_ observations are also recommendable for high-salinity waters. Ideally, a reevaluation of the calibration functions should be performed using only data from natural seawater samples.

The existence of five approaches that show inconsistencies among them discourages widespread [CO_3_^2–^]_spec_ determination. The ocean CO_2_ community would benefit from an SOP and the availability of CRMs for [CO_3_^2–^]_spec_ measurement. The SOP should include well-described and validated best practices to be implemented unambiguously by other users interested in setting up [CO_3_^2–^]_spec_ measurements.

## Supporting Information Available

Appendixes showing (A) Global ocean distribution of carbonate ion content and saturation states. Figure S1. (B) Evolution of the methodology for measuring carbonate ion content, Figures S2 and S3 and Tables S1 and S2. (C) Terminology, Table S3. (D) Δ[CO_3_^2–^] uncertainty assessment: uncertainty in [CO_3_^2–^]_calc_ estimation, Figure S4 and Tables S4 and S5. (E) Δ[CO_3_^2–^] uncertainty assessment: [CO_3_^2–^]_spec_ uncertainty related to the absorbance ratio. Monte Carlo analysis, Figure S5. (F) Δ[CO_3_^2–^] uncertainty assessment: [CO_3_^2–^]_spec_ uncertainty related to the absorbance ratio. Absorbance measurements with both Pb(II) reagents and different spectrophotometers, Figures S6 and S7 (PDF)

## Abbreviations

BK800: Beckman DU800 spectrophotometer
BY08: approach for measuring [CO_3_^2–^]_spec_ described by Byrne and Yao^24^
CO_2_: carbon dioxide
[CO_3_^2–^]_calc_: calculated carbonate ion content from pH-TA or DIC-TA pairs
[CO_3_^2–^]_spec_: spectrophotometric carbonate ion content
DIC: dissolved inorganic carbon
EAS13: approach for measuring [CO_3_^2–^]_spec_ described by Easley et al.^28^
GOA-ON: Global Ocean Acidification Observing Network
OA: ocean acidification
PAT15: approach for measuring [CO_3_^2–^]_spec_ described by Patsavas et al.^29^
PE850: PerkinElmer Lambda 850 spectrophotometer
*R*: absorbance ratio
SHA17: approach for measuring [CO_3_^2–^]_spec_ described by Sharp et al.^30^
SHA19: approach for measuring [CO_3_^2–^]_spec_ described by Sharp and Byrne^25^
SHI2401: Shimadzu UV2401 spectrophotometer
SHI2600: Shimadzu UV2600 spectrophotometer
TA: Total alkalinity
