# Supplementary material for: Spectrophotometric Measurement of Carbonate Ion in Seawater over a Decade: Dealing with Inconsistencies
Source: Environ Sci Technol. 2022 Jun 7;56(12):7381–95. doi: 10.1021/acs.est.1c06083 (PMC9228043; doi:10.1021/acs.est.1c06083)
Supplement: Supplementary file 1 — es1c06083_si_001.pdf [file es1c06083_si_001.pdf]

SUPPORTING INFORMATION for:

Spectrophotometric measurement of carbonate ion in seawater along a decade: dealing with inconsistencies

Elisa F. Guallart[*,1,2], Noelia M. Fajar[1,3], Maribel I. García-Ibáñez[4,2], Mónica Castaño-Carrera[1], Rocío Santiago-Doménech[5], Abed El Rahman Hassoun[6,7], Fiz F. Pérez[3], Regina A. Easley[8], Marta Álvarez[1]

[1] Centro Oceanográfico de A Coruña (COAC-IEO), CSIC, DC 15001 A Coruña, Spain

[2] Institut de Ciències del Mar (ICM), CSIC, DC 08003 Barcelona, Spain

[3] Instituto de Investigacións Mariñas (IIM), CSIC, DC 36208 Vigo, Spain

[4] School of Environmental Sciences, University of East Anglia (UEA), Norwich NR47TJ, United Kingdom

[5] Centro Oceanográfico de Baleares (COB-IEO), CSIC, DC 07015 Palma de Mallorca, Balearic Islands, Spain

[6] GEOMAR Helmholtz Centre for Ocean Research Kiel, D- 24105 Kiel, Germany

[7] National Center for Marine Sciences, National Council for Scientific Research in Lebanon (CNRS-L), Beirut, Lebanon.

[8] Chemical Sciences Division, National Institute of Standards and Technology (NIST), MS 8391, Gaithersburg, MD 20899, United States

Corresponding author:

**e-mail: efguallart@icm.csic.es*; phone (+34) 932309500; fax: (+34)932309555.

Number of pages: 24

Number of figures: 7

Number of tables: 5

Appendix A: Global ocean distribution of carbonate ion content and saturation states.

The saturation state (Ω) of calcium carbonate ($CaCO_3$) minerals is defined as:

$$\Omega = [Ca^{2+}]\,[CO_3^{2-}] / K_{sp} \quad (S1)$$

where $[CO_3^{2-}]$ is the carbonate ion content, $[Ca^{2+}]$ is the calcium content, and K_{sp} is the apparent stoichiometric solubility product for a given $CaCO_3$ mineral phase, calcite or aragonite. The solubility of both compounds increases slightly at lower temperature and strongly with increasing pressure. Since $[Ca^{2+}]$ is closely proportional to salinity, Ω is largely determined by in situ $[CO_3^{2-}]$ variations with regard to in situ $[CO_3^{2-}]$ saturation. The depth at which $CaCO_3$ minerals, aragonite and calcite, are in equilibrium is known as the saturation depth or saturation horizon (Ω = 1). There is a pronounced shoaling of the saturation horizons of both minerals from the Atlantic to the Indian and then to the Pacific oceans. This occurs because of the lower total alkalinity (TA) to dissolved inorganic carbon (DIC) ratio (TA/DIC) in the intermediate and deep waters of the latter two oceans relative to the Atlantic. Among oceans, the aragonite saturation horizon is always shallower than the calcite saturation horizon due to its higher solubility.[1,2]

In the surface ocean, warmer and saltier regions show higher $[CO_3^{2-}]$ and are more saturated with respect to $CaCO_3$ minerals than colder and less saline regions (Figure S1A and B). Evaporation (precipitation) processes that increase (decrease) salinity levels cause a TA and DIC increase (reduction) in a 2:1 ratio.[3] High TA/DIC waters with a higher buffer capacity present, accordingly, high values of $[CO_3^{2-}]$ and Ω for aragonite, $\Omega_{aragonite}$ (Figure S1A and B). In addition, the buffer factor ($-\omega_{DIC}$; in $mmol \cdot kg^{-1}$)[4] showing the fractional change in $\Omega_{aragonite}$ for changes in DIC when TA is constant, decreases as the TA/DIC ratio increases (Figure S1C). Therefore, for a given increase in DIC due to anthropogenic carbon uptake, the decrease in Ω is higher for high TA/DIC waters.

The lower row panels in Figure S1 show the same variables as the upper panels: in situ $[CO_3^{2-}]$, $\Omega_{aragonite}$, and $-\omega_{DIC}$ for the full-depth merged dataset reported in this study, evidencing the broad range of oceanic conditions covered with regard to the global ocean ranges.

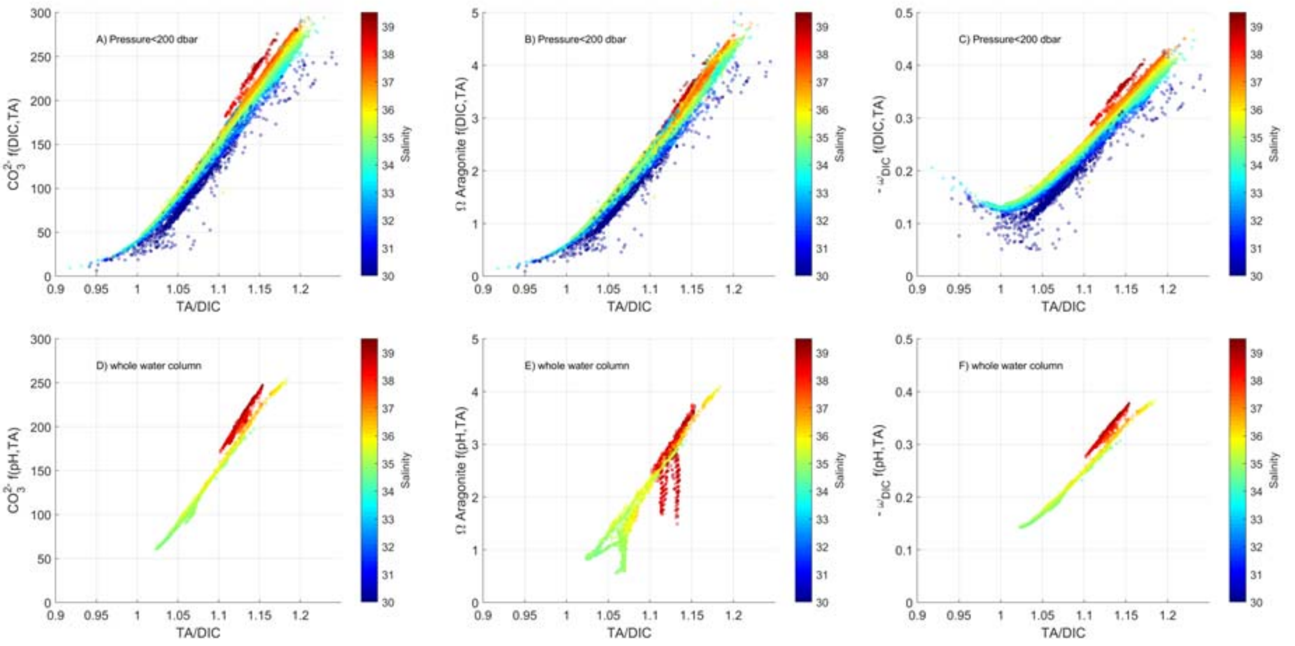

Figure S1. Distribution of (A and D) in situ [CO_3^{2-}] (CO_3^{2-}; in µmol·kg^{-1}), (B and E) in situ aragonite saturation ($\Omega_{aragonite}$; dimensionless), and (C and F) in situ buffer factor ($-\omega_{DIC}$; in mmol·kg^{-1}) as a function of the TA to DIC ratio (TA/DIC). Upper panels show calculated variables from the global surface ocean (pressure < 200 dbar) DIC and TA data from GLODAPv2.2019.[5] Lower panels show the calculated variables from full water column pH and TA from the merged dataset in this study (Table 1). See Section 2.2 regarding thermodynamic CO_2 calculations. The z-axis shows salinity in color scale**.**

Appendix B: Evolution of the methodology for measuring carbonate ion content.

The formation of lead carbonate ($PbCO_3^0$) in seawater is given by:

$$Pb^{2+} + CO_3^{2-} \leftrightarrow PbCO_3^0 \quad (S2)$$

The formation (or stability) constant for $PbCO_3^0$, ${}_{CO3}\beta_1$, refers to the equilibrium constant for the formation of $PbCO_3^0$ in solution and is a measure of the strength of the interaction between $[Pb^{2+}]_T$ and $[CO_3^{2-}]_T$ to form $PbCO_3^0$. It is given by:

$$_{CO3}\beta_1 = \frac{[PbCO_3^0]}{[Pb^{2+}]_T[CO_3^{2-}]_T} \quad (S3)$$

where $[Pb^{2+}]_T$ represents the total amount of content of free Pb^{2+}, Pb(II)-chloride species and minor amounts of Pb(II)-sulfate species. $[CO_3^{2-}]_T$ is the total amount of content of free and ion-paired carbonate and $[PbCO_3^0]$ represents the total amount of content of Pb(II) complexed with carbonate, including contributions from mixed ligand species. All amounts of substance content are given in $\mu mol \cdot kg^{-1}$ of seawater.

When Pb(II) complexes with $[CO_3^{2-}]_T$ in Pb(II)-enriched seawater, the ultraviolet spectrum of the solution is affected and $[CO_3^{2-}]_T$ can be determined spectrophotometrically ($[CO_3^{2-}]_{spec}$) through Equation (1) in the main manuscript. This equation allows for the direct determination of $[CO_3^{2-}]_{spec}$ in terms of the ratio of Pb(II) absorbances of the sample at two given wavelengths (R value; Equation (2)), provided that the remaining terms in Equation (1) ($\log\{_{CO3}\beta_1/e_2\}$, e_1, and e_3/e_2) have been accurately characterized.

Table S1 summarizes the main features of the five different approaches that have been described for the determination of $[CO_3^{2-}]_{spec}$ since 2008, as the methodology has been periodically revised, here abbreviated as BY08,[6] EAS13,[7] PAT15,[8] SHA17,[9] and SHA19.[10] Table S1 reports the different conditions at which the terms $\log\{_{CO3}\beta_1/e_2\}$, e_1, and e_3/e_2 in Equation (1) were characterized for each approach. In fact, the characterization of these terms is implicitly a calibration because it relates a given R value to a particular $[CO_3^{2-}]_T$ and, thus, the existence of various sets of calibration functions, one per approach, that yield five different $[CO_3^{2-}]_{spec}$ values from a single R measurement, is in practice equivalent to having five different methods for measuring $[CO_3^{2-}]_{spec}$. In this regard, the five approaches overall followed the same fitting procedure for calibrating the spectrophotometric terms in Equation (1) that consists of making R measurements in seawater over a range of conditions (e.g., salinity and temperature) where the seawater has been independently analyzed for at least two of the usually measured CO_2 system parameters (e.g., pH, TA, and DIC). It is then possible to calculate a value for $[CO_3^{2-}]_T$ ($[CO_3^{2-}]_{calc}$) from the application of a given equilibrium model for seawater, taking account of all acid-base systems occurring in the sample, and seeking to fit the terms $\log\{_{CO3}\beta_1/e_2\}$, e_1, and e_3/e_2 as

functions of salinity and temperature. Table S2 summarizes the reported calibration functions for log{$_{CO3}\beta_1$/e$_2$}, e_1, and e_3/e_2 in Equation (1) for each of the five approaches as a function of salinity (BY08, EAS13, PAT15, and SHA17; all values referred to 25 ºC) and also temperature (SHA19). All formulations are referred to atmospheric pressure.

The successive reformulations of the calibration functions (Table S2) have, in turn, been fostered by successive reviews and upgrades of the methodological procedure proposed to obtain accurate enough values of R. Note that any change in the procedure to determine R implicitly forces the reformulation of the calibration equations that relate R values with given amounts of $[CO_3^{2-}]_{calc}$. In this regard, R values are affected by (Table 1): i) the Pb(II) reagent, ii) the spectrophotometer used, and iii) additional corrections to readjust measured R values into "closer to the true" values (Equations (4) and (5)). On the other hand, the $[CO_3^{2-}]_{calc}$ data used can also impact the fitting of the calibration functions, through (Table 1): i) the thermodynamic model of seawater assumed (i.e., CO_2 system constants and the other constants of known acid-base systems), ii) the quality of the measurements of the two CO_2 systems parameters used as input for obtaining $[CO_3^{2-}]_{calc}$ (pH, TA, and DIC) and iii) the ranges of $[CO_3^{2-}]$ studied (i.e., the region of study and the use of natural versus laboratory-modified seawater).

BY08 characterized the first set of calibration functions for the terms log{$_{CO3}\beta_1$/e$_2$}, e_1, and e_3/e_2 in Equation (1) (Table S2). They constrained the corresponding terms experimentally in seawater modified in the laboratory and characterized the Pb(II) absorbance response (R values; Equation (2)) by using lead chloride ($PbCl_2$) (Table S1).

EAS13 reviewed the approach of BY08 using natural seawater samples and pH values measured spectrophotometrically with purified dye (Table 1). EAS13 found significant biases in the $[CO_3^{2-}]$ residuals (observations with respect to calculated values) obtained with their approach at $[CO_3^{2-}]_{calc} \geq 150$ µmol·kg^{-1}, evidencing the underestimation of $[CO_3^{2-}]_{spec}$ measurements at higher amounts of content. EAS13 related those results to a possible lack of applicability of the Pb(II) complexation model (Equation S2) at high pH values,[11] suggesting that it should likely be extended to the formation of the $Pb(CO_3)_2^{2-}$ species.

The review of the methodology reported by PAT15 proposed a change in the reagent used for characterizing the R values, using lead perchlorate ($Pb(ClO_4)_2$) instead of $PbCl_2$ to increase the signal-to-noise ratio of the absorbance measurements. This change also doubled the final Pb(II) concentration in the cuvette with regard to former protocols (Table S1). Consequently, PAT15 proposed an additional correction for readjusting the measured R data (Equation (4)) because of the perturbation of the sample due to the Pb(II) reagent addition. PAT15 refitted the calibration functions (Table S2) according to new field data, with the overall aim of improving the determination of $[CO_3^{2-}]_{spec}$ well above 180 µmol·kg^{-1}. PAT15 did assume another

thermodynamic model for $[CO_3^{2-}]_{calc}$ with regard to BY08 and EAS13 (Table S1). The authors kept using the e_3/e_2 term described by BY08 and proposed new values for $\log\{_{CO3}\beta_1/e_2\}$ and e_1 (Table S2). The approach by PAT15 yielded $[CO_3^{2-}]_{spec}$ that were accurate enough over a large range of amount of carbonate content (about 75 µmol·kg^{-1} – 260 µmol·kg^{-1}) but the authors stated that further work would be required to assess the applicability of the new Pb(II) reagent and the refitted calibration functions for low-carbonate (i.e., at lower salinity) waters. PAT15 attributed the observed residuals between observations and calculated values to the underlying chemical measurements of the input parameters for estimating $[CO_3^{2-}]_{calc}$, not to differences in the fitting protocols for characterizing the calibration functions, nor to the potential lack of applicability of the Pb(II) complexation model at high pH values.[11]

The reviews by EAS13 and PAT15 aimed mainly to improve the parametrization of the complexation between $[Pb^{2+}]_T$ and $[CO_3^{2-}]_T$ by refitting the calibration functions of the terms in Equation (1) into values representative of natural seawater by enlarging the range of ocean conditions, mainly salinity, assessed to fit them (Table S1). The following review of the methodology by SHA17 deviates from this rationale and suggests that carbonate residuals between observations and calculated values are mainly attributable to the spectrophotometer equipment through potential calibration offsets of the particular spectrophotometer used for measuring the absorbances needed for obtaining the *R* data. Uncalibrated equipment might produce artifacts in the UV signal at the measured wavelengths, generating incongruent *R* and $[CO_3^{2-}]_{spec}$ values. The authors also used $Pb(ClO_4)_2$ for measuring *R*. In SHA17, no perturbation correction (Equation (4)) was recommended. Instead, they reported an equation for correcting measured *R* as a function of a wavelength offset term (Equation (5)) (Table 1). SHA17 also recharacterized the calibration functions (Table S2). Additionally, the authors recommended recording Pb(II) absorbances at wavelengths surrounding the primary target wavelengths (e.g., 233 nm, 234 nm, and 235 nm) because of the use of multi-wavelength measurement techniques in the future.

Finally, SHA19 reported the most recent review of the calibration functions. The authors did not change the procedure to obtain R^0 with regard to SHA17(Table S2) but extended the characterization of the terms over a broader range of salinity and temperature to enable in situ observations by combining field datasets from former works with laboratory data (Table S1).

Table S1. Summary of fitting conditions for the characterization of the terms log{$_{CO3}\beta_1$/e$_2$}, e_1, and e_3/e_2 in Equation (1) for the five approaches of the spectrophotometric technique to measure carbonate ion in seawater abbreviated as BY08,[6] EAS13,[7] PAT15,[8] SHA17,[9] and SHA19[10]. SW stands for seawater. $pH_{electrode}$ stands for seawater pH measured with an electrode calibrated on the total scale in natural seawater whose pH was determined with thymol blue dye, and pH_{pur} stands for seawater pH on the total scale measured with purified m-cresol dye. DIC stands for dissolved inorganic carbon, and TA for total alkalinity, both in µmol·kg^{-1}. S stands for salinity, t for temperature in ºC, $[CO_3^{2-}]_{calc}$ is the amount of carbonate ion content calculated from paired measured CO_2 variables, K_1 and K_2 stand for the first and second CO_2 equilibrium constants, K_{HSO4} for the bisulfate constant, K_B for the boric acid constant and TB for the total boron-to-chlorinity ratio. R corresponds to de absorbance ratio of Pb(II) at two wavelengths (Equation (2)). R^0 corresponds to the initial ratio before perturbation of the sample due to Pb(II) reagent addition for PAT15 (Equation (4)), and to the ratio corrected for wavelength offsets of the spectrophotometer for SHA17 and SHA19. $\Delta\lambda_{241.1}$ is the spectrophotometer-specific wavelength offset at λ = 241.10 nm, defined as the wavelength location of a holmium oxide standard absorbance peak as specified by the manufacturer minus the wavelength at which the spectrophotometer reports the peak, which causes a reversal in the sign of Equation (5) with regard to SHA17. The $[CO_3^{2-}]_{spec}$ precision (in µmol·kg^{-1} or %) and the $[CO_3^{2-}]_{spec}$ standard uncertainty (in %) are reported for each method approach.

Table S1	BY08	EAS13	PAT15	SHA17	SHA19
**Pb(II) reagent**	$PbCl_2$	$PbCl_2$	$Pb(ClO_4)_2$	$Pb(ClO_4)_2$	$Pb(ClO_4)_2$
**[Pb(II); μmol·L^{-1}]** in 10cm pathlength cuvette	7.5	7.5	15.2	15.2	15.2
**Region of study**	-	US West Coast and Arctic	US East Coast and Gulf of Mexico	US East and West coasts	As in PAT15 and SHA17
**Laboratory data**	yes (modified Gulf of Mexico SW)	no	no	no	yes (modified Gulf of Mexico SW)
**Measured CO_2 system variables**	$pH_{electrode}$, TA	pH_{pur}, DIC, TA	pH_{pur}, DIC, TA	pH_{pur}, DIC	pH_{pur}, DIC, TA
**$[CO_3^{2-}]_{calc}$ range** in μmol·kg^{-1}	$68<[CO_3^{2-}]_{calc}<400$	$38<[CO_3^{2-}]_{calc}<258$	$73<[CO_3^{2-}]_{calc}<258$	$68<[CO_3^{2-}]_{calc}<258$	As in PAT15 and SHA17
***S* range**	20<S<36	26.6<S<34.9	28.6<S<36.6	34.50<S<36.72	20<S<40
**CO_2 system and other constants used for $[CO_3^{2-}]_{calc}$**	K_1&K_2 Mehrbach et al.[12] as refit by Dickson and Millero;[13] K_{HSO4}, K_B and TB not reported	K_1&K_2 Mehrbach et al. [12] as refit by Dickson and Millero;[13] K_{HSO4} Dickson,[14a] K_B Dickson,[15a] TB not reported	K_1&K_2 Lueker et al.,[16] K_{HSO4} Dickson,[14] TB Lee et al. [17]	As in PAT15	As in PAT15
***t* range**	25 ºC ± 0.05 ºC	25 ºC ± 0.05 ºC	25 ºC ± 0.05 ºC	25 ºC ± 0.05 ºC	3 ºC < *t* ± 0.05 ºC < 40 ºC
**Spectrophotometer**	HP Agilent 8453	HP Agilent 8453	HP Agilent 8453	HP Agilent 8453	HP Agilent 8453
**Pb(II) reagent addition correction**	not evaluated	evaluated, no correction applied	evaluated, correction applied $\log(R-R^0)= -17.6664R^2+19.8995R-7.7324$	evaluated, no correction applied	evaluated, no correction applied
**Wavelength offset correction**	not identified	not identified	not identified	identified $R^0 = R + 0.0265 \times \Delta\lambda_{241.1}$	As in SHA17
**$[CO_3^{2-}]_{spec}$ precision,** in μmol·kg^{-1} or %	not reported	± 1.7, ± 2.28 %	not reported	± 1.9 μmol·kg^{-1}	± 0.7 %
**$[CO_3^{2-}]_{spec}$ standard uncertainty** in %	± 2% or less	± 2.3 %	± 2.1 %	± 1.5 %	± 2 %

[a] K_{HSO4} and KB in EAS13 added as R. Easley personal communication

Table S2. Summary of the coefficients for the spectrophotometric terms in Equation (1) to calculate $[CO_3^{2-}]_{spec}$ in seawater according to the five different approaches (Table S1). Each parameter is expressed with the general equation form of *Z*, following Sharp and Byrne.[10] The different approaches are denoted as BY08,[6] EAS13,[7] PAT15,[8] SHA17,[9] and SHA19.[10] S is salinity and t is temperature in ºC. Formulations by BY08, EAS13, PAT15, and SHA17 are referred to 25 ºC, being only salinity-dependent. The approach by SHA19 is also temperature-dependent (Table S1). All coefficients apply to a pressure of 1 atm.

$$Z = a_0 + b_0 S + b_1 S^2 + C_0 t + C_1 t^2 + d_0 St$$

Z		$a_0 \cdot 10$	$b_0 \cdot 10^3$	$b_1 \cdot 10^4$	$c_0 \cdot 10^3$	$c_1 \cdot 10^5$	$d_0 \cdot 10^5$
**log(β/e₂)**	BY08	60.87	-84.95	9.36	-	-	-
	EAS13	55.13	-53.58	5.166	-	-	-
	PAT15	55.07074	-41.259	1.8	-	-	-
	SHA17	68.7057	-142.142	19.0892	-	-	-
	SHA19	55.6674	-51.0194	4.61423	-	-	-13.6998
**e1**	BY08	2.215	-0.5554	0.844	-	-	-
	EAS13	2.293	-0.5554	0.844	-	-	-
	PAT15	3.11907	-2.396	0.8	-	-	-
	SHA17	7.87458	-33.9648	5.83574	-	-	-
	SHA19	1.09519	4.49666	-	1.95519	2.4446	-2.01796
**e3/e2**	BY08	30.61	-87.3	9.363	-	-	-
	EAS13	30.91	-87.3	9.363	-	-	-
	PAT15	30.61	-87.3	9.363	-	-	-
	SHA17	25.2288	-38.3205	-	-	-	-
	SHA19	32.4812	-79.7676	6.28521	-11.8691	-3.58709	32.5849

The calibration functions that describe log{$_{CO3}\beta_1$/e$_2$}, e_1, and e_3/e_2) in Equation (1) seem to have come up with differing values for them (Table S2). To better compare the effect of the differing values for these terms on $[CO_3^{2-}]_{spec}$, Equation (1) in the main manuscript can be algebraically transformed to express $[CO_3^{2-}]_{spec}$ in μmol·kg^{-1} as follows:

$$[CO_3^{2-}]_{spec} = \left[10^6 * 10^{\log\left[\frac{CO3\beta_1}{e_2}\right]}\right] * \left[\frac{1}{R - e_1}\right] * \left[1 - R * \frac{e_3}{e_2}\right] \quad (S4)$$

Figures S2 and S3 show the terms between brackets in Equation (S4) for the different approaches. The inset in Figure S2A shows $[CO_3^{2-}]_{spec}$ at 25 ºC and salinity 35 as a function of R, which clearly increase as R decreases. For the same conditions in temperature and salinity, $[CO_3^{2-}]_{spec}$ as a function of R by EAS13 is about 5 % higher than by BY08 at $[CO_3^{2-}]_{spec} > 150$ μmol.kg^{-1} or $R < 0.46$, and about 5 % lower for higher R values (Figure S2A). PAT15, SHA17, and SHA19 approaches return $[CO_3^{2-}]_{spec}$ values higher than BY08 for the whole R range, being larger than 10 % for $[CO_3^{2-}]_{spec} > 150$ μmol·kg^{-1} ($R < 0.46$), particularly with the PAT15 approach (Figure S2A).

The first term in Equation (S4), $\left[10^6 * 10^{\log\left[\frac{CO3\beta_1}{e_2}\right]}\right]$ (in μmol·kg^{-1}), is only marginally dependent on temperature when using SHA19 formulations, being mostly dependent on salinity for all the approaches (Table S2) and increases with it (inset in Figure S2B). This is directly linked to the presence of more carbonate ion content in higher salinity waters (Figure S1). This first term in Equation (S4) is similar for BY08 and EAS13, with EAS13 being slightly lower (Figure S2B). However, in PAT15, SHA17, and SHA19 the relation with salinity differs with regard to BY08 and also between them across the salinity range. Results among approaches are close between them at about ± 5 % only at salinity around 37 ± 1, except for SHA19.

This first term in Equation (S4) $\left[10^6 * 10^{\log\left[\frac{CO3\beta_1}{e_2}\right]}\right]$ is modulated upwards by $\left[\frac{1}{R-e_1}\right]$ and downwards by $\left[1 - R * \frac{e_3}{e_2}\right]$ (Figure S3). Regarding these two terms, the main changes in the evolution of the methodology relate to the $\left[\frac{1}{R-e_1}\right]$ term (upper row in Figure S3), which is related to higher carbonate ion content (lower R values), where more remarkable inconsistencies between spectrophotometric and calculated carbonate ion content have been found.[7–10,18] In this regard, the greatest changes were introduced by PAT15 and SHA19 (Figure S3C and S3E) in low salinity waters, while SHA17 introduced the changes for high salinity waters (Figure S3D). The term $\left[1 - R \cdot \frac{e_3}{e_2}\right]$ most strongly influences carbonate ion calculations at low carbonate content (Figure S3F-J) and has slightly changed with regard to BY08 overall. PAT15 reported the same coefficients for this term, being SHA17 and SHA19 who introduced the greatest modifications compared to BY08.

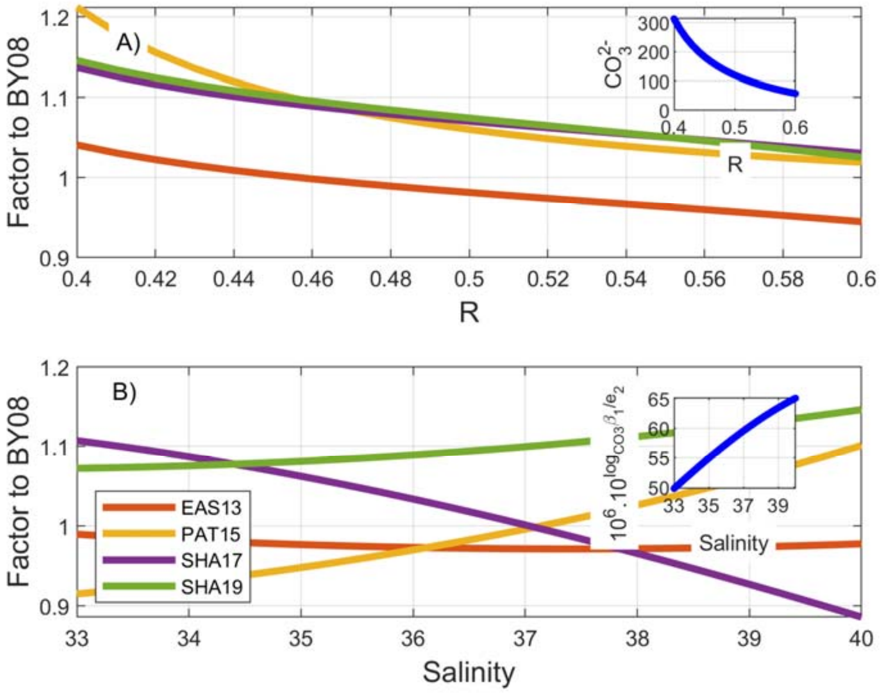

Figure S2. Comparison of the methodology approaches by EAS13, PAT15, SHA17, and SHA19 (Table S1) shown as factors with respect to the first approach by BY08: (A) $[CO_3^{2-}]_{spec}$ reported at 25 ºC and salinity 35 as a function of the Pb(II) absorbance ratio (R; Equation (2)), and (B) the term $\left[10^6 \cdot 10^{\log[\frac{CO3\beta_1}{e_2}]}\right]$ in Equation (S4) as a function of salinity. The corresponding insets in each panel show absolute results with the BY08 approach (both in µmol·kg^{-1}; in blue).

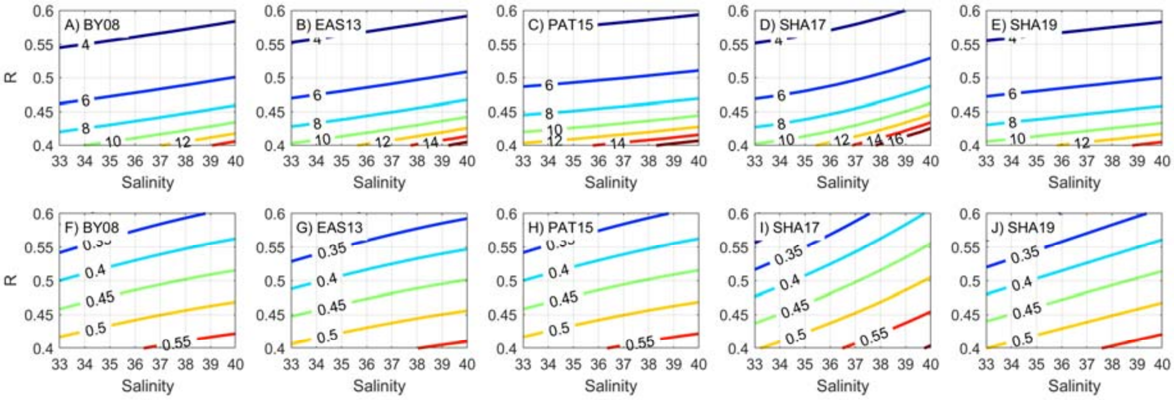

Figure S3. Comparison of the terms $[1/(R - e_1)]$ (upper row) and $[1 - R \cdot e_3/e_2]$ (lower row) in Equation (S4) for the five approaches in Table S1 as a function of the absorbance ratio (R; Equation (2)) and salinity. The corresponding formulations are in Table S2. All axes are dimensionless.

Appendix C. Terminology

Terminology relative to measurement of CO_2 parameters

Accuracy. It refers to how close a measurement is to the correct (i.e., true) value for that measurement. Accuracy of CO_2 measurements is assessed against Certified Reference Materials (CRMs), if available. In the case of TA and DIC, there are CRMs[19] to assess the accuracy of the respective measurements. pH and $[CO_3^{2-}]_{spec}$ lack CRMs and their accuracy can only be examined against the respective calculated values, from the AT and DIC certified values of a CRM. In the case of pH, Tris buffer is also used as a standard material to compare against, although it is not considered a reference material.

Analytical Precision. It refers to how close is the agreement between repeated measurements that are repeated under the same conditions (i.e., the spread of the measured values one to another). In this study, precision is assessed as the standard deviation of a number of repeated measurements.

Total standard uncertainty. It is an estimate of how much your measured values deviate from a standard or expected value (i.e., the amount by which the measurement differs from the expected value). If measurements are not accurate or precise, then the uncertainty of the measurements will be very high. Sources of uncertainty can have either a random or a systematic origin. For $[CO_3^{2-}]_{spec}$, SHA19 described in detail the distinction between the random and systematic components of standard uncertainty;

The random uncertainty refers to the analytical precision of the measurements. SHA19 estimated it at ± 0.7 % (Table S1).

The systematic uncertainty refers to the uncertainty inherent to the fitting of the calibration functions (Table S2), which is based on $[CO_3^{2-}]_{calc}$ (Appendix B). This component of uncertainty can cause a bias in $[CO_3^{2-}]_{spec}$ due to the set of functions used. SHA19 estimated it at ±1.9 % in their approach.

The resulting combined (random plus systematic) total standard uncertainty assigned to $[CO_3^{2-}]_{spec}$ measurements amounts to ±2 % in SHA19, being consistent with previous approaches (Table S1). Hence, the total $[CO_3^{2-}]_{spec}$ standard uncertainty considers both the measurement imprecision and uncertainty inherent to the calibration functions.

Terminology relative to datasets description

In this study, dataset description applies to carbonate ion residuals shown in the results section.

Dispersion. Is a way of describing how scattered is a set of data. It refers to the variability or scatter of the data; when it is large, the data are widely scattered, while when it is small,

the data are clustered. Dispersion of data can be measured as the interquartile range (i.e. the difference between the 3rd and the 1st quartiles of the data; Figure 2) or as standard deviation (Table S4 and S5), among other measures of dispersion.

The dispersion of carbonate ion residuals is related to the random component of $[CO_3^{2-}]_{spec}$ uncertainty, through the photometric accuracy of the spectrophotometer used for measurement.

Bias. It refers to the observed trend in carbonate ion residuals with regard to $[CO_3^{2-}]_{calc}$. $[CO_3^{2-}]_{calc}$ residuals can be biased towards positive or negative values, within or beyond the ± 4 % limit for internal consistency.

The bias in carbonate ion residuals is related to the systematic component of $[CO_3^{2-}]_{spec}$ uncertainty, through the wavelength accuracy of the spectrophotometer used for measurement.

Terminology relative to measurement of absorbance

The spectrophotometer specifications (Table S3) impact the random and systematic components of $[CO_3^{2-}]_{spec}$ uncertainty, through the measurement of *R* (Equation (2)) values.

Table S3. Technical specifications of the spectrophotometer models used in the determination of $[CO_3^{2-}]_{spec}$ during the cruises shown in Table 1. Specifications of the spectrophotometer model (last column) used in the bibliography defining the $[CO_3^{2-}]_{spec}$ approaches (Table S1) are also shown. The estimated absorbance ratio random uncertainty (R random uncertainty), according to the photometric accuracy, is also shown. Photometric accuracy is in absorbance units (*A*).

	**Shimadzu UV2401 (SHI2401)**	**Shimadzu UV2600 (SHI2600)**	**Beckman DU800 (BK800)[a]**	**Perkin Elmer Lambda 850 (PE850)**	**Agilent UV8453**
Wavelength accuracy (nm)	± 0.3	± 0.3	± 0.5	± 0.08	± 0.5
Wavelength repeatability (nm)	± 0.1	± 0.05	± 0.2	± 0.02	± 0.02
Photometric Accuracy (*A*)	± 0.004	± 0.003	± 0.005	± 0.003	± 0.005
Stray Light (%)	< 0.015	< 0.005	< 0.05	< 0.00007	< 0.05
R random uncertainty	± 0.008	± 0.006	± 0.01	± 0.006	± 0.01

[a]All spectrophotometers are double beam except Beckman DU800 that is single beam. The Agilent UV8453 spectrophotometer, (Table S1) is also single beam.

Isosbestic point. In this study, it refers to the particular wavelength, in nanometers (nm), at which Pb(II) absorbance spectra shows the same value of absorbance at different conditions (i.e., at different [CO_3^{2-}]). This is, the value of absorbance does not depend on [CO_3^{2-}].

Photometric accuracy. It marks the ability of the equipment to discern absorbance values within limits of confidence. It is a source of random uncertainty in *R*. A spectrophotometer with low photometric accuracy will introduce more random noise in *R* measurements, resulting in more dispersed *R* measurements. Table S3 shows the value of *R* random uncertainty for each spectrophotometer, according to their respective photometric accuracy.

Wavelength accuracy. It settles the capability of the equipment to read the absorbance at a given wavelength. It is a source of systematic uncertainty in *R*. This parameter can impact the accuracy of *R* measurements with regard to their true values and, thus, the goodness of the fitting of the set of functions (Table S2) due to the use of inaccurate *R* values. As reported in the Introduction and Appendix B, SAH17 proposed a correction to account for systematic offsets in *R* data (Equation (5)).

Wavelength repeatability. It is the stability of wavelength measurement. A measured wavelength should not drift within a range of wavelengths; it must be stable within a specified wavelength repeatability.

Stray light. This parameter introduces an error in the recorded absorbance, leading to negative deviations in Beer-Lambert's Law, causing increasing deviations as absorbance increases. It affects the signal-to-noise ratio, causing increasing photometric underestimation as absorbance increases. It is a source of random uncertainty in *R*.

Appendix D. $\Delta[CO_3^{2-}]$ uncertainty assessment: uncertainty in $[CO_3^{2-}]_{calc}$ estimation.

As described in Section 2.2 in the main text, the five sets of calibration functions (Table S2) reported for the determination of $[CO_3^{2-}]_{spec}$ in seawater were assessed through the study of the magnitude and distribution of carbonate ion residuals ($\Delta[CO_3^{2-}]$). In this regard, $[CO_3^{2-}]_{calc}$ are considered the reference or true values. The underlying reason for this is that the $[CO_3^{2-}]_{spec}$ methodology is itself defined according to $[CO_3^{2-}]_{calc,}$ through the characterization of the calibration functions that are fitted against $[CO_3^{2-}]_{calc}$, as explained in Appendix B.

The definition of $\Delta[CO_3^{2-}]$ ($[CO_3^{2-}]_{spec}$ - $[CO_3^{2-}]_{calc}$) in this study is the same as in EAS13, but opposite to BY08, PAT15, SHA17 and SHA19. $\Delta[CO_3^{2-}]$ are studied with regard $[CO_3^{2-}]_{calc}$, evaluating whether $[CO_3^{2-}]_{spec}$ are overestimated or underestimated regarding $[CO_3^{2-}]_{calc}$. Thus, $\Delta[CO_3^{2-}]$ indicate how close are $[CO_3^{2-}]_{spec}$ to the expected $[CO_3^{2-}]_{calc}$ values and, thus, how good is the performance of a set of calibration functions. Until a scientific agreement is achieved on what methodological approach is the best to measure the most accurate $[CO_3^{2-}]_{spec}$ and until a specific CRM will be available for this parameter, the five $[CO_3^{2-}]_{spec}$ obtained among approaches should be considered all potentially true values to be examined against $[CO_3^{2-}]_{calc}$ for accuracy.

With regard to $[CO_3^{2-}]_{calc}$ in this study, the following remarks should be considered:

1- Differences in $[CO_3^{2-}]_{calc}$ obtained from either the pH-TA and DIC-TA input pairs are small (blue dots in Figure S4), showing absolute values with a mean and standard deviation of 1.9 $\mu mol\cdot kg^{-1} \pm 1.5\ \mu mol\cdot kg^{-1}$. As reported in the main text, all the results shown in this study were obtained with $[CO_3^{2-}]_{calc}$ from pH-TA.

2- The influence of the seawater model assumed for CO_2 system calculations, which relies on the different thermodynamic options for K_1 and K_2, K_{HSO4} and TB constants assumed for calculating $[CO_3^{2-}]_{calc}$, is small when the pH-TA input pair is considered, within 1 $\mu mol\cdot kg^{-1}$ (cyan dots in Figure S4). The two sets of thermodynamic constants tested with the CO2SYS package for MATLAB are (A) K_1K_2=10 (Lueker et al.[16]) and K_{HSO4} = 3 (Dickson[14] and Lee et al.[17]), and (B) K_1K_2 = 4 (Mehrbach et al.[12] as refit by Dickson and Millero[13]) and K_{HSO4} = 1 (Dickson[14] and Uppström[20]). Only these two sets of functions were used since these were the sets of constants used to assess $[CO_3^{2-}]_{calc}$ in the five evolving approaches of the methodology, as shown in Table S1.

3- If $[CO_3^{2-}]_{calc}$ total uncertainty is reevaluated by readjusting measured pH values to pH values that would have been obtained using purified dye, according to Liu et al.[21] (data for Sigma Aldrich, in their Figure 2), it increases by 0.8 % – 1.5 % over the $[CO_3^{2-}]_{calc}$ study range. Hence, the use of unpurified dye for pH measurements in the merged dataset in this study is not relevant for the interpretation and discussion of the reported results.

4 - The total uncertainty propagated in $[CO_3^{2-}]_{calc}$ through using the software *errors*, from Orr et al.,[22] is proportional to the concentration itself. It ranges between 2.5 µmol·kg^{-1} and 4.5 µmol·kg^{-1} for $[CO_3^{2-}]_{calc}$ obtained with the DIC-TA pair (black dots in Figure S4), and between 2.5 µmol·kg^{-1} and 8 µmol·kg^{-1} for the pH-TA pair (red dots in Figure S4) within the $[CO_3^{2-}]_{calc}$ study range (68 µmol·kg^{-1} – 252 µmol·kg^{-1}), so from 3.7 % to 1.8 % with DIC-TA and from 3.7 % to 3.2 % with pH-TA.

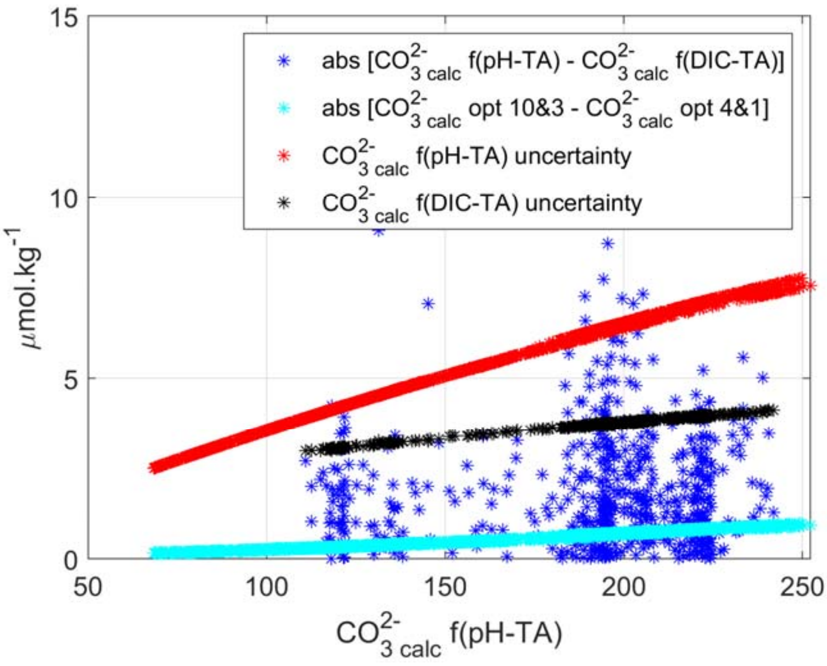

Figure S4. Sources of uncertainty in the calculated carbonate ion content ($[CO_3^{2-}]_{calc}$, in µmol·kg^{-1}) based on the dataset of this study. All data are shown as absolute values. Blue symbols show the difference in $[CO_3^{2-}]_{calc}$ between data estimated with the pH-TA and DIC-TA input pairs. The cyan symbols depict the range of differences between $[CO_3^{2-}]_{calc}$ obtained from the pH-TA pair using two thermodynamic options in the CO2SYS package for MATLAB[23] [K_1K_2 = 10 (Lueker et al.[16]) and K_{HSO4} = 3 (Dickson[14] & Lee et al.[17]) and K_1K_2 = 4 (Mehrbach et al.,[12] as refit by Dickson and Millero[13]) and K_{HSO4} = 1 (Dickson[14] and Uppström[20])]. The black and red symbols depict the combined standard uncertainty in $[CO_3^{2-}]_{calc}$ estimated from DIC-TA and pH-TA input pairs, respectively, based on the *errors* script by Orr et al.[22] [standard uncertainties: 0.01 for pH, ± 3 µmol·kg^{-1} for DIC or TA (Section 2.1) and those in Table 1 in Orr et al.[22] for the constants].

Mean $\Delta[CO_3^{2-}]$ and standard deviation for each cruise in this study are shown in Table S4 (for the whole range of amount of $[CO_3^{2-}]_{calc}$ content) and Table S5 (for low, medium, and high ranges of amount of $[CO_3^{2-}]_{calc}$ content). Note that Tables S4 and S5 and the respective captions are shown in separated pages due to space reasons.

Table S4. Mean ± standard deviation values of $\Delta[CO_3^{2-}]$ ($\Delta[CO_3^{2-}] = [CO_3^{2-}]_{spec} - [CO_3^{2-}]_{calc}$; in $\mu mol \cdot kg^{-1}$) for each of the cruises in Table 1. The number of samples averaged is shown in parenthesis. The cruise alias and the spectrophotometer (SHI2401, SHI2600, PE850, or BK800; Table S3) used are indicated in the column headers. $[CO_3^{2-}]_{spec}$ is calculated with five different formulations: BY08, EAS13, PAT15, SHA17, and SHA19 (Table S2); and $\Delta[CO_3^{2-}]$ from data measured with $PbCl_2$ or $Pb(ClO_4)_2$ are shown separately. $[CO_3^{2-}]_{calc}$ is calculated with pH and TA. Calculations are at 25 ºC and 1 atm except for SHA19 that is at 1 atm and the exact temperature of analysis. The SHA17 and SHA19 approaches include a wavelength correction (Δλ; Table S1) that equals 0.2 nm for cruises where the SHI2600 was used (HOTMIX, MSM72, RADPROF, RADCOR, and iFADO; Table 1) and is null for the remaining cases. $\Delta[CO_3^{2-}] < 4$ %, 4 % < $\Delta[CO_3^{2-}] < 10$ %, or $\Delta[CO_3^{2-}] > 10$ % are highlighted in green, orange and red, respectively.

Table S5. Mean ± standard deviation values of $\Delta[CO_3^{2-}]$ ($\Delta[CO_3^{2-}] = [CO_3^{2-}]_{spec} - [CO_3^{2-}]_{calc}$; in $\mu mol \cdot kg^{-1}$) for each of the cruises in Table 1 for three different $[CO_3^{2-}]_{calc}$ ranges: $[CO_3^{2-}]_{calc} <$ 100 $\mu mol \cdot kg^{-1}$, 100 < $[CO_3^{2-}]_{calc} <$ 180 $\mu mol \cdot kg^{-1}$, and $[CO_3^{2-}]_{calc} >$ 180 $\mu mol \cdot kg^{-1}$, shown in the upper, middle and lower lines within each cell, respectively. The number of samples is shown in parenthesis. The cruise alias and the spectrophotometer (SHI2401, SHI2600, PE850, or BK800; Table S3) used are indicated in the column headers. $[CO_3^{2-}]_{spec}$ is calculated with five different formulations: BY08, EAS13, PAT15, SHA17, and SHA19 (Table S2); and $\Delta[CO_3^{2-}]$ from data measured with $PbCl_2$ or $Pb(ClO_4)_2$ are shown separately. $[CO_3^{2-}]_{calc}$ is calculated with pH and TA. Calculations are reported at 25 ºC and 1 atm except for SHA19 that is reported at 1 atm and the exact temperature of analysis. The SHA17 and SHA19 approaches include a wavelength correction (Δλ; Table S1) that equals 0.2 nm for cruises where the SHI2600 was used (HOTMIX, MSM72, RADPROF, RADCOR, and iFADO; Table 1) and is null for the remaining cases. $\Delta[CO_3^{2-}] < 4$ %, 4 % < $\Delta[CO_3^{2-}] < 10$ %, or $\Delta[CO_3^{2-}] > 10$ % are highlighted, in green, orange and red, respectively.

Table S4

		CAIBOX PE850	MOC2 SHI2401	HOTMIX SHI2600	OVIDE PE850	TALPRO BK800	MEDWAVES PE850	MSM72 SHI2600	RADPROF SHI2600	RADCOR SHI2600	iFADO SHI2600	iFADO 2 PE850
**BY08**	$PbCl_2$	4.2 ± 2.7 (272)	1 ± 4 (625)	-4.7 ± 5.6 (321)	0.6 ± 2.8 (196)				4.4 ± 3.7 (48)	0.8 ± 6.2 (127)	1.9 ± 1.5 (28)	-4.5± 3.1 (33)
	$Pb(ClO_4)_2$					-5.4 ± 11.6 (111)	-12.8 ± 3.6 (294)	-12.8 ± 3.6 (294)	-1.0 ± 2.7 (44)	1.4 ± 5.7 (72)	-4.6 ± 3.3 (32)	-11.2 ± 3.3 (34)
**EAS13**	$PbCl_2$	2.5 ± 2.7 (272)	-0.8 ± 3.5 (625)	-5.4 ± 5.4 (321)	-1.4 ± 2.9 (196)				2.6 ± 4.1 (48)	0.3 ± 6.2 (127)	-0.1 ± 1.8 (28)	-6.4 ± 3.1 (33)
	$Pb(ClO_4)_2$					-6.3 ± 12.1 (111)	-21.3 ± 6.2 (147)	-13.8 ± 3.5 (294)	-3.1 ± 2.9 (44)	0.2 ± 5.6 (72)	-6.8 ± 3 (32)	-13.3 ± 2.8 (34)
**PAT15**	$PbCl_2$	24.4 ± 7.7 (272)	17.4 ± 10.4 (625)	33.9 ± 7.9 (321)	17.2 ± 4.9 (196)				22.9 ± 6.3 (48)	26.2 ± 7.7 (127)	20 ± 5.6 (28)	14.2 ± 5.4 (33)
	$Pb(ClO_4)_2$					35.0 ± 14.4 (111)	-0.4 ± 3.2 (147)	27.7 ± 4.7 (294)	16.2 ± 4.9 (44)	25.0 ± 5.9 (72)	12.2 ± 3.2 (32)	5.8 ± 2.4 (34)
**SHA17**	$PbCl_2$	16.2 ± 5.1 (272)	10.6 ± 6.2 (625)	9.4 ± 4.4 (321)	10.4 ± 3.5 (196)				8.8 ± 4.1 (48)	7.2 ± 6.5 (127)	6.1 ± 2.4 (28)	6.5 ± 3.8 (33)
	$Pb(ClO_4)_2$					20.6 ± 13.9 (111)	-8.1 ± 3.6 (147)	2.5 ± 3.6 (294)	2.9 ± 2.9 (44)	7.3 ± 5.6 (72)	-0.8 ± 2.6 (32)	-1.2 ± 1.4 (34)
**SHA19**	$PbCl_2$	20.3 ± 5.7 (272)	12.6 ± 6.7 (625)	12.6 ± 6.7 (625)	11.4 ± 3.8 (196)				10.8 ± 4.2 (48)	9.8 ± 5.9 (127)	7.3 ± 2.5 (28)	6.0 ± 3.8 (33)
	$Pb(ClO_4)_2$					22.6 ± 13.5 (111)	-7.4 ± 3.4 (147)	0.7 ± 3.1 (294)	4.8 ± 3.1 (44)	8.6 ± 4.6 (72)	0.1 ± 2.4 (32)	-1.7 ± 1.7 (34)

Table S5		CAIBOX<br>PE850	MOC2<br>SHI2401	HOTMIX<br>SHI2600	OVIDE<br>PE850	TALPRO<br>BK800	MEDWAVES<br>PE850	MSM72<br>SHI2600	RADPROF<br>SHI2600	RADCOR<br>SHI2600	iFADO<br>SHI2600	iFADO 2<br>PE850
**BY08**	$PbCl_2$	---------------<br>4.5 ± 2.4 (236)<br>2.7 ± 4.1 (36)	2.3 ± 1.7 (238)<br>1.6 ± 3.2 (299)<br>-4.3 ± 6.3 (88)	---------------<br>1.8 ± 2.5 (47)<br>-5.8 ± 5.3 (274)	---------------<br>0.7 ± 2.7 (188)<br>-2.4 ± 3.3 (8)				---------------<br>4.3 ± 3.8 (45)<br>6.1 ± 3.1 (3)	---------------<br>1.4 ± 5.3 (98)<br>-1.1 ± 8.1 (29)	---------------<br>1.8 ± 1.5 (25)<br>2.6 ± 1.6 (3)	---------------<br>-4 ± 2.5 (26)<br>-6.3 ± 4.5 (7)
	$Pb(ClO_4)_2$					---------------<br>---------------<br>5.4 ± 11.6 (111)	---------------<br>-16 ± 4.0 (114)<br>-28.4 ± 3.4 (33)	---------------<br>-9.9 ± 2.7 (3)<br>-12.9 ± 3.6 (291)	---------------<br>-1 ± 2.7 (41)<br>-1.9 ± 1.7 (3)	---------------<br>2.3 ± 5.2 (62)<br>-4.6 ± 4.6 (10)	---------------<br>-3.6 ± 1.6 (28)<br>-11.7 ± 3.7 (4)	---------------<br>9.8 ± 1.8 (27)<br>-16.4 ± 2.3 (7)
**EAS13**	$PbCl_2$	---------------<br>2.4 ± 2.4 (236)<br>3.2 ± 4.2 (36)	-0.5 ± 1.7 (238)<br>-0.7 ± 3.3 (299)<br>-1.9 ± 6.5 (88)	---------------<br>-0.4 ± 2.6 (47)<br>-6.2 ± 5.2 (274)	---------------<br>-1.4 ± 2.9 (188)<br>-2.2 ± 3.6 (8)				---------------<br>2.3 ± 4 (45)<br>6.6 ± 3.3 (3)	---------------<br>0.6 ± 5.5 (98)<br>-0.6 ± 8.3 (29)	---------------<br>-0.4 ± 1.5 (25)<br>2.6 ± 1.6 (3)	---------------<br>-6.3 ± 2.5 (26)<br>-6.7 ± 4.9 (7)
	$Pb(ClO_4)_2$					---------------<br>---------------<br>6.3 ± 12.1 (111)	---------------<br>-18.7 ± 3.9 (114)<br>-30.3 ± 3.5 (33)	---------------<br>-11.9 ± 3.2 (3)<br>-13.8 ± 3.5 (291)	---------------<br>-3.2 ± 2.9 (41)<br>-1.9 ± 1.7 (3)	---------------<br>1.0 ± 5.3 (62)<br>-4.6 ± 4.8 (10)	---------------<br>-6 ± 1.8 (28)<br>-12.4 ± 3.9 (4)	---------------<br>-12.3 ± 1.9 (27)<br>-17.3 ± 2.3 (7)
**PAT15**	$PbCl_2$	---------------<br>22 ± 4.3 (236)<br>40.0 ± 6.2 (36)	10.7 ± 2.2 (238)<br>16.3 ± 4.5 (299)<br>39.5 ± 8.2 (88)	---------------<br>19.7 ± 4.6 (47)<br>36.3 ± 5.4 (274)	---------------<br>16.7 ± 4.2 (188)<br>29.0 ± 4.5 (8)				---------------<br>21.9 ± 5.0 (45)<br>38.1 ± 4.1 (3)	---------------<br>25.5 ± 6.7 (98)<br>28.8 ± 10.1 (29)	---------------<br>18.4 ± 3.1 (25)<br>33.3 ± 1.5 (3)	---------------<br>12.1 ± 2.7 (26)<br>21.9 ± 6.2 (7)
	$Pb(ClO_4)_2$					---------------<br>---------------<br>35.0 ± 14.4 (111)	---------------<br>-0.7 ± 2.6 (114)<br>0.4 ± 4.8 (33)	---------------<br>20.4 ± 0.6 (3)<br>27.8 ± 4.7 (291)	---------------<br>15.3 ± 3.8 (41)<br>27.9 ± 2.2 (3)	---------------<br>25.2 ± 5.9 (62)<br>23.8 ± 5.9 (10)	---------------<br>11.8 ± 2.9 (28)<br>14.9 ± 4.5 (4)	---------------<br>5.0 ± 1.3 (27)<br>9.2 ± 2.7 (7)
**SHA17**	$PbCl_2$	---------------<br>14.8 ± 3.2 (236)<br>25.4 ± 5.3 (36)	6.9 ± 2 (238)<br>10.2 ± 3.7 (299)<br>22 ± 7.2 (88)	---------------<br>6 ± 2.7 (47)<br>10.0 ± 4.4 (274)	---------------<br>10.1 ± 3.3 (188)<br>16.3 ± 4 (8)				---------------<br>8.4 ± 3.9 (45)<br>14.4 ± 3.3 (3)	---------------<br>7.4 ± 5.7 (98)<br>6.5 ± 8.7 (29)	---------------<br>5.6 ± 1.8 (25)<br>10.6 ± 1.6 (3)	---------------<br>5.4 ± 2.3 (26)<br>10.5 ± 5.5 (7)
	$Pb(ClO_4)_2$					---------------<br>---------------<br>20.6 ± 13.9 (111)	---------------<br>-7.2 ± 2.8 (114)<br>-11.4 ± 4.2 (33)	---------------<br>-0.4 ± 1.1 (3)<br>2.5 ± 3.6 (291)	---------------<br>2.7 ± 2.8 (41)<br>5.8 ± 1.7 (3)	---------------<br>8 ± 5.4 (62)<br>2.6 ± 5 (10)	---------------<br>-0.2 ± 1.8 (28)<br>-4.9 ± 3.9 (4)	---------------<br>-1.2 ± 1.0 (27)<br>-1.2 ± 2.5 (7)
**SHA19**	$PbCl_2$	---------------<br>18.6 ± 3.5 (236)<br>31.5 ± 4.1 (36)	8.1 ± 2 (238)<br>12.3 ± 3.9 (299)<br>25.5 ± 6.4 (88)	---------------<br>6.0 ± 2.9 (47)<br>8.6 ± 4.3 (274)	---------------<br>11.1 ± 3.4 (188)<br>18.3 ± 5 (8)				---------------<br>10.3 ± 3.8 (45)<br>18.0 ± 3.4 (3)	---------------<br>9.1 ± 5.4 (98)<br>12.3 ± 7 (29)	---------------<br>6.7 ± 1.7 (25)<br>12.7 ± 2.1 (3)	---------------<br>4.9 ± 2.8 (26)<br>9.9 ± 4.8 (7)
	$Pb(ClO_4)_2$					---------------<br>---------------<br>22.6 ± 13.5 (111)	---------------<br>-6.5 ± 2.7 (114)<br>-10.3 ± 4.0 (33)	---------------<br>0.6 ± 1.9 (3)<br>0.7 ± 3.1 (291)	---------------<br>4.5 ± 2.9 (41)<br>9.3 ± 1.7 (3)	---------------<br>9.1 ± 4.5 (62)<br>5.3 ± 3.8 (10)	---------------<br>0.6 ± 1.9 (28)<br>-3.4 ± 2.9 (4)	---------------<br>-1.6 ± 1.3 (27)<br>-1.8 ± 2.9 (7)

Appendix E. $\Delta[CO_3^{2-}]$ uncertainty assessment: $[CO_3^{2-}]_{spec}$ uncertainty related to the absorbance ratio. Monte Carlo analysis.

The random uncertainty inherent to the methodology for $[CO_3^{2}]_{spec}$ determination was tested with a Monte Carlo analysis. To examine the minimum random errors that could be ascribed to *R* measurements, the value of ±0.006 was selected to perform the corresponding perturbations, because it is the lowest value for *R* uncertainty derived from the respective photometric accuracy specifications of all spectrophotometers used in this study (Table S3). It derives from PE850 and SHI2600, in particular.

The Monte Carlo analysis modifies *R* measurements according to a random value from a normal distribution with a mean of zero and a standard deviation of 0.006/2. The perturbed *R* values are used with the EAS13 approach to obtain $[CO_3^{2}]_{spec}$ perturbed values that are used to calculate $\Delta[CO_3^{2-}]$. Figure S5 shows the corresponding results for *R* data measured with $PbCl_2$, highlighting that $\Delta[CO_3^{2-}]$ have larger random uncertainty at higher values of $[CO_3^{2-}]_{calc}$, particularly at $[CO_3^{2-}]_{calc} > 180$ µmol·kg^{-1}. The same results were found for data measured with $Pb(ClO_4)_2$ (data not shown). Consequently, even higher random uncertainty would be expected for *R* data measured with the remaining spectrophotometers (BK800 and SHI2401), including the model used for defining the spectrophotometric parameters in Equation (S4), UV8453, since all of them have higher uncertainty in photometric accuracy than PE850 and SHI2600 (Table S3).

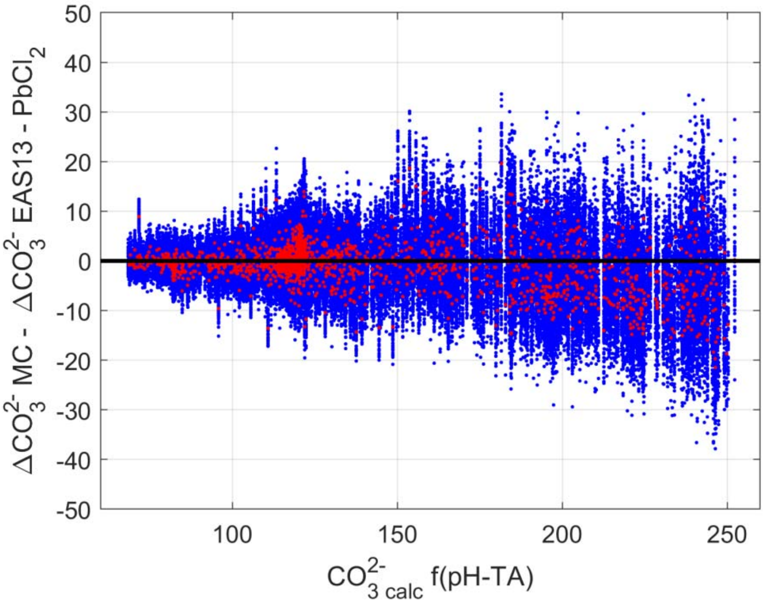

Figure S5. Red dots correspond to $[CO_3^{2-}]_{spec}$ obtained with the EAS13 approach minus $[CO_3^{2-}]_{calc}$ ($\Delta[CO_3^{2-}]$-EAS13 = $[CO_3^{2-}]_{spec}$ - $[CO_3^{2-}]_{calc}$; in µmol·kg^{-1}) as a function of $[CO_3^{2-}]_{calc}$ (calculated from pH and TA), for all $PbCl_2$ data among cruises in Table 1. Blue dots correspond to a Monte Carlo analysis showing the difference between $\Delta[CO_3^{2-}]$-MC and $\Delta[CO_3^{2-}]$-EAS13. $\Delta[CO_3^{2-}]$-MC correspond to perturbed $[CO_3^{2-}]_{spec}$ determined with the EAS13 approach (but on *R* data perturbed introducing random errors of ±0.006) minus $[CO_3^{2-}]_{calc}$ (in µmol·kg^{-1}).

Appendix F: $\Delta[CO_3^{2-}]$ uncertainty assessment: $[CO_3^{2-}]_{spec}$ uncertainty related to the absorbance ratio. Absorbance measurements with both Pb(II) reagents and different spectrophotometers.

During the RADPROF and iFADO cruises, discrete *R* values were measured on replicate samples, using $PbCl_2$ and $Pb(ClO_4)_2$ to obtain $[CO_3^{2-}]_{spec}$ with the SHI2600 (Table 1). During the iFADO cruise, both SHI2600 and PE850 were used with the two reagents for measuring discrete *R* values. Additionally, during the iFADO cruise, scan readings from 220 nm to 370 nm were performed in quadruplicate samples. Using both reagents and SHI2600 and PE850, three to five scans per sample were recorded with sampling intervals of 0.5 nm or 1 nm, in different exercises, and averaged to obtain a mean and standard deviation absorbance value by wavelength. Mean and standard deviation absorbances were obtained at discrete target wavelengths, λ, from each mean scan by averaging values about ± 2 nm from the target λ: ${}_{234}A$, ${}_{250}A$ and ${}_{350}A$. Corresponding results by reagent and spectrophotometer are shown in Figures S6 and S7.

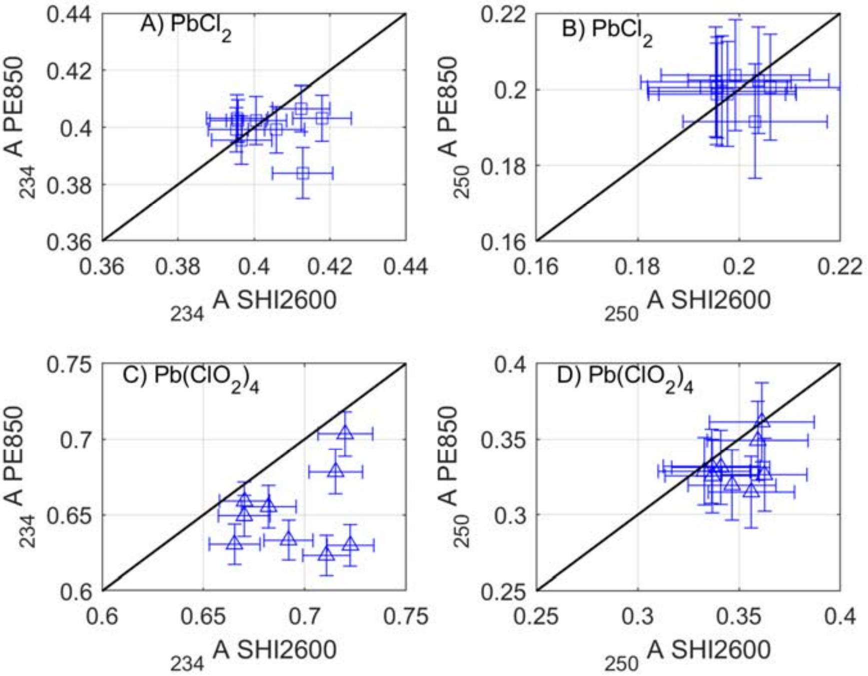

Figure S6. Comparison of Pb(II) absorbance between the SHI2600 and the PE850 spectrophotometers at (A, C) λ= 234 nm and (B, D) λ= 250 nm wavelengths for sample replicates measured using $PbCl_2$ (upper panels) and $Pb(ClO_4)_2$ (lower panels) during the iFADO cruise. Absorbance readings at 234 nm and 250 nm are corrected for the background absorbance at 350 nm. Points and error bars correspond to the mean and standard deviations of absorbance measurements about ± 2 nm from the target λ values.

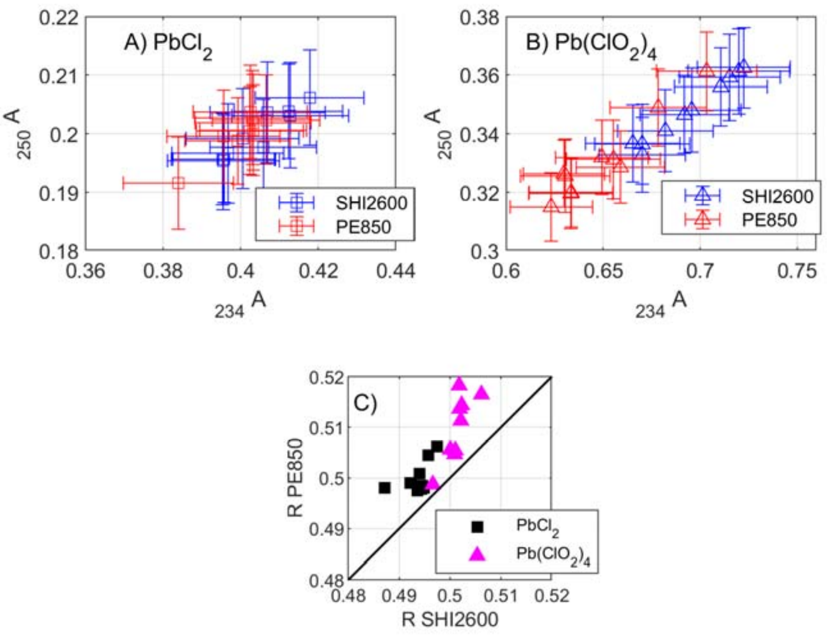

Figure S7. Relationship between Pb(II) absorbance at λ = 234 nm and λ = 250 nm wavelengths for sample replicates measured with the SHI2600 (in blue) and the PE850 (in red) spectrophotometers, using (A) $PbCl_2$ and (B) $Pb(ClO_4)_2$ during the iFADO cruise. Both absorbance readings are corrected for the background absorbance at 350 nm. Points and error bars correspond to the mean and standard deviations of absorbance measurements about ± 2 nm from the target λ values. (C) Comparison of *R* values ($R = [({}_{250}A - {}_{350}A)/({}_{234}A - {}_{350}A)]$) between spectrophotometers using $PbCl_2$ (black squares) and $Pb(ClO_4)_2$ (pink triangles).

REFERENCES

(1) Chung, S.-N.; Park, G.-H.; Lee, K.; Key, R. M.; Millero, F. J.; Feely, R. A.; Sabine, C. L.; Falkowski, P. G. Postindustrial Enhancement of Aragonite Undersaturation in the Upper Tropical and Subtropical Atlantic Ocean: The Role of Fossil Fuel CO_2. *Limnology and Oceanography* **2004**, *49* (2), 315–321. https://doi.org/10.4319/lo.2004.49.2.0315.

(2) Jiang, L.-Q.; Feely, R. A.; Carter, B. R.; Greeley, D. J.; Gledhill, D. K.; Arzayus, K. M. Climatological Distribution of Aragonite Saturation State in the Global Oceans. *Global Biogeochemical Cycles* **2015**, *29* (10), 1656–1673. https://doi.org/10.1002/2015GB005198.

(3) Zeebe, R. E.; Wolf-Gladrow, D. *CO_2 in Seawater: Equilibrium, Kinetics, Isotopes*; Elsevier Science, 2001; Vol. 65.

(4) Egleston, E. S.; Sabine, C. L.; Morel, F. M. M. Revelle Revisited: Buffer Factors That Quantify the Response of Ocean Chemistry to Changes in DIC and Alkalinity. *Global Biogeochemical Cycles* **2010**, *24* (1). https://doi.org/10.1029/2008GB003407.

(5) Olsen, A.; Lange, N.; Key, R. M.; Tanhua, T.; Álvarez, M.; Becker, S.; Bittig, H. C.; Carter, B. R.; Cotrim da Cunha, L.; Feely, R. A.; van Heuven, S.; Hoppema, M.; Ishii, M.; Jeansson, E.; Jones, S. D.; Jutterström, S.; Karlsen, M. K.; Kozyr, A.; Lauvset, S. K.; Lo Monaco, C.; Murata, A.; Pérez, F. F.; Pfeil, B.; Schirnick, C.; Steinfeldt, R.; Suzuki, T.; Telszewski, M.; Tilbrook, B.; Velo, A.; Wanninkhof, R. GLODAPv2.2019 – an Update of GLODAPv2. *Earth Syst. Sci. Data* **2019**, *11* (3), 1437–1461. https://doi.org/10.5194/essd-11-1437-2019.

(6) Byrne, R. H.; Yao, W. Procedures for Measurement of Carbonate Ion Concentrations in Seawater by Direct Spectrophotometric Observations of Pb(II) Complexation. *Marine Chemistry* **2008**, *112* (1), 128–135. https://doi.org/10.1016/j.marchem.2008.07.009.

(7) Easley, R. A.; Patsavas, M. C.; Byrne, R. H.; Liu, X.; Feely, R. A.; Mathis, J. T. Spectrophotometric Measurement of Calcium Carbonate Saturation States in Seawater. *Environ. Sci. Technol.* **2013**, *47* (3), 1468–1477. https://doi.org/10.1021/es303631g.

(8) Patsavas, M. C.; Byrne, R. H.; Yang, B.; Easley, R. A.; Wanninkhof, R.; Liu, X. Procedures for Direct Spectrophotometric Determination of Carbonate Ion Concentrations: Measurements in US Gulf of Mexico and East Coast Waters. *Marine Chemistry* **2015**, *168*, 80–85. https://doi.org/10.1016/j.marchem.2014.10.015.

(9) Sharp, J. D.; Byrne, R. H.; Liu, X.; Feely, R. A.; Cuyler, E. E.; Wanninkhof, R.; Alin, S. R. Spectrophotometric Determination of Carbonate Ion Concentrations: Elimination of Instrument-Dependent Offsets and Calculation of In Situ Saturation States. *Environ. Sci. Technol.* **2017**, *51* (16), 9127–9136. https://doi.org/10.1021/acs.est.7b02266.

(10) Sharp, J. D.; Byrne, R. H. Carbonate Ion Concentrations in Seawater: Spectrophotometric Determination at Ambient Temperatures and Evaluation of Propagated Calculation Uncertainties. *Marine Chemistry* **2019**, *209*, 70–80. https://doi.org/10.1016/j.marchem.2018.12.001.

(11) Easley, R. A.; Byrne, R. H. The Ionic Strength Dependence of Lead (II) Carbonate Complexation in Perchlorate Media. *Geochimica et Cosmochimica Acta* **2011**, *75* (19), 5638–5647. https://doi.org/10.1016/j.gca.2011.07.007.

(12) Mehrbach, C.; Culberson, C. H.; Hawley, J. E.; Pytkowicx, R. M. Measurement of the Apparent Dissociation Constants of Carbonic Acid in Seawater at Atmospheric Pressure. *Limnology and Oceanography* **1973**, *18* (6), 897–907. https://doi.org/10.4319/lo.1973.18.6.0897.

(13) Dickson, A. G.; Millero, F. J. A Comparison of the Equilibrium Constants for the Dissociation of Carbonic Acid in Seawater Media. *Deep Sea Research Part A. Oceanographic Research Papers* **1987**, *34* (10), 1733–1743. https://doi.org/10.1016/0198-0149(87)90021-5.

(14) Dickson, A. G. Standard Potential of the Reaction: AgCl(s) + $12H_2$(g) = Ag(s) + HCl(Aq), and and the Standard Acidity Constant of the Ion HSO_4^- in Synthetic Sea Water from 273.15 to 318.15 K. *The Journal of Chemical Thermodynamics* **1990**, *22* (2), 113–127. https://doi.org/10.1016/0021-9614(90)90074-Z.

(15) Dickson, A. G. Thermodynamics of the Dissociation of Boric Acid in Synthetic Seawater from 273.15 to 318.15 K. *Deep Sea Research Part A. Oceanographic Research Papers* **1990**, *37* (5), 755–766. https://doi.org/10.1016/0198-0149(90)90004-F.

(16) Lueker, T. J.; Dickson, A. G.; Keeling, C. D. Ocean $_PCO_2$ Calculated from Dissolved Inorganic Carbon, Alkalinity, and Equations for K_1 and K_2: Validation Based on Laboratory Measurements of CO_2 in Gas and Seawater at Equilibrium. *Marine Chemistry* **2000**, *70* (1), 105–119. https://doi.org/10.1016/S0304-4203(00)00022-0.

(17) Lee, K.; Kim, T.-W.; Byrne, R. H.; Millero, F. J.; Feely, R. A.; Liu, Y.-M. The Universal Ratio of Boron to Chlorinity for the North Pacific and North Atlantic Oceans. *Geochimica et Cosmochimica Acta* **2010**, *74* (6), 1801–1811. https://doi.org/10.1016/j.gca.2009.12.027.

(18) Fajar, N. M.; García-Ibáñez, M. I.; SanLeón-Bartolomé, H.; Álvarez, M.; Pérez, F. F. Spectrophotometric Measurements of the Carbonate Ion Concentration: Aragonite Saturation States in the Mediterranean Sea and Atlantic Ocean. *Environ. Sci. Technol.* **2015**, *49* (19), 11679–11687. https://doi.org/10.1021/acs.est.5b03033.

(19) Dickson, A. G.; Afghan, J. D.; Anderson, G. C. Reference Materials for Oceanic CO_2 Analysis: A Method for the Certification of Total Alkalinity. *Marine Chemistry* **2003**, *80* (2), 185–197. https://doi.org/10.1016/S0304-4203(02)00133-0.

(20) Uppström, L. R. The Boron/Chlorinity Ratio of Deep-Sea Water from the Pacific Ocean. *Deep Sea Research and Oceanographic Abstracts* **1974**, *21* (2), 161–162. https://doi.org/10.1016/0011-7471(74)90074-6.

(21) Liu, X.; Patsavas, M. C.; Byrne, R. H. Purification and Characterization of Meta-Cresol Purple for Spectrophotometric Seawater PH Measurements. *Environ. Sci. Technol.* **2011**, *45* (11), 4862–4868. https://doi.org/10.1021/es200665d.

(22) Orr, J. C.; Epitalon, J.-M.; Dickson, A. G.; Gattuso, J.-P. Routine Uncertainty Propagation for the Marine Carbon Dioxide System. *Marine Chemistry* **2018**, *207*, 84–107. https://doi.org/10.1016/j.marchem.2018.10.006.

(23) Van Heuven, S.; Pierrot, D.; Rae, J. W. B.; Lewis, E.; Wallace, D. W. R. MATLAB Program Developed for CO_2 System Calculations. *ORNL/CDIAC-105b* **2011**, *530*.
